# Oncogenic K-Ras upregulates *ITGA6* expression via FOSL1 to induce anoikis resistance and synergizes with αV-Class integrins to promote EMT

**DOI:** 10.1038/onc.2017.177

**Published:** 2017-06-12

**Authors:** K Zhang, S-M Myllymäki, P Gao, R Devarajan, V Kytölä, M Nykter, G-H Wei, A Manninen

**Affiliations:** 1 Biocenter Oulu, Centre of Excellence in Cell-Extracellular Matrix Research, Faculty of Biochemistry and Molecular Medicine, University of Oulu, Oulu, Finland; 2 Prostate Cancer Research Center, Institute of Biomedical Technology and BioMediTech, University of Tampere and Tampere University Hospital, Tampere, Finland

## Abstract

In many cancer types, integrin-mediated signaling regulates proliferation, survival and invasion of tumorigenic cells. However, it is still unclear how integrins crosstalk with oncogenes to regulate tumorigenesis and metastasis. Here we show that oncogenic K-Ras^V12^ upregulates α6-integrin expression in Madin–Darby canine kidney (MDCK) cells via activation of the mitogen-activated protein kinase/extracellular signal-regulated kinase (ERK)/Fos-related antigen 1-signaling cascade. Activated α6-integrins promoted metastatic capacity and anoikis resistance, and led to perturbed growth of MDCK cysts. Transcriptomic analysis of K-Ras^V12^-transformed MDCK cells also revealed robust downregulation of αV-class integrins. Re-expression of αV-integrin in K-Ras^V12^-transformed MDCK cells synergistically upregulated the expression of Zinc finger E-box-binding homeobox 1 and Twist-related protein 1 and triggered epithelial-mesenchymal transition leading to induced cell motility and invasion. These results delineate the signaling cascades connecting oncogenic K-Ras^V12^ with α6- and αV-integrin functions to modulate cancer cell survival and tumorigenesis, and reveal new possible strategies to target highly oncogenic K-Ras^V12^ mutants.

## Introduction

Aberrant integrin-mediated cell–extracellular matrix (ECM) signaling can contribute to the abnormal growth and morphology of cancer cells.^1, 2, 3^ Polarized epithelial cells form extensive cell–cell contacts (tight junctions, adherens junctions and desmosomes) and cell–ECM contacts (focal adhesions and hemi-desmosomes), all of which contribute to establishment of apical, lateral and basal membrane domains each with distinct protein composition.^4, 5^ Formation and maintenance of these polarized domains and contacts is critical for regulating not only cell shape but also cell growth, differentiation and survival. Therefore, it is not surprising that loss of polarized organization within epithelial cancer tissues correlates with the aggressiveness of the disease.^6^ Moreover, pre-tumorigenic lesions can be formed by interfering with the functions of cell polarity proteins, suggesting that polarity proteins also serve a tumor suppressor function.^7^ In line with these findings, polarized organization of surrounding epithelial cells can suppress oncogenic properties of tumor cells.^8, 9^ These studies have shown that some but not all oncogenes have the ability to escape suppression from the polarized environment when surrounded by normal epithelial cells.^9^ How this is regulated is still unclear.

The best-known examples of dual functions of polarity proteins come from components of cell–cell adhesion complexes. E-cadherin at adherent’s junctions is frequently lost in invasive cancers.^10^ In addition, E-cadherin targeting to adherens junctions leads to stimulated growth.^11^ Similarly, cell–ECM interactions are critical for cancer cell proliferation and invasion, but these interactions are also complex and likely to be context dependent. Integrins are important ECM receptors, which convey signals from the ECM into cells to regulate and maintain epithelial cell growth, survival and polarity.^5, 12, 13^ However, the specific integrin heterodimers involved and the exact molecular mechanisms remain uncertain. Non-canonical integrin-mediated signaling is often reported in cancers.^1, 2, 3, 14^

Transformed cancer cells can escape epithelial monolayer via extrusion to apical or basolateral side.^15^ Although abnormal growth signaling might allow survival of apically extruded tumor cells without ECM contact, basolateral extrusion is generally thought to promote potentiate spread and invasion of tumor cells and eventually promote formation of metastatic lesions.^10^ Integrins are ideally positioned to convey signals and functions required for escape of oncogenic cells from polarized epithelium. Here we report that K-Ras^V12^/ mitogen-activated protein kinase (MAPK)/extracellular signal-regulated kinase (ERK)/Fos-related antigen 1 (FOSL1)-signaling cascade activates α6-integrin expression, leading to anoikis resistance and increased metastatic potential of K-Ras^V12^-transformed cells. K-Ras^V12^ transformation also led to downregulation of αV-class integrins in Madin–Darby canine kidney (MDCK) cells that are considered to be a model for normal epithelial cells. We show that re-expression αV-integrin in K-Ras^V12^-MDCK cells is sufficient to convert them into highly invasive mesenchymal cells. This conversion was mediated via autocrine activation of transforming growth factor (TGF)-β signaling pathway leading to activation of epithelial-mesenchymal transition (EMT) transcription factors Zinc finger E-box-binding homeobox 1 (ZEB1), TWIST1 and Snail2. Taken together, our findings demonstrate important and novel insight into the signaling cascades connecting oncogenic K-Ras^V12^ with α6- and αV-integrin functions to modulate cancer cell survival and tumorigenesis, and reveal new possible strategies to target highly oncogenic K-Ras^V12^ mutants.

## Results

### Oncogenic K-Ras^V12^ transforms MDCK cells to enable their extrusion and overcome tumor suppression by the surrounding normal epithelium

**Figure 1:**
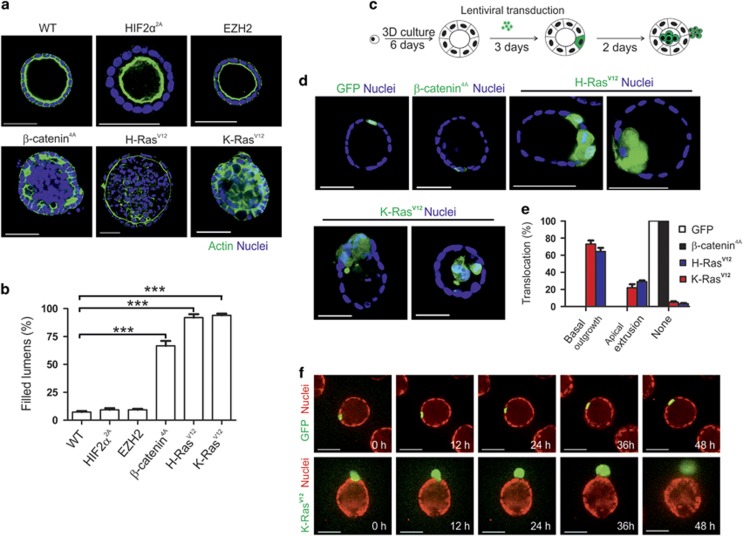
K-Ras^V12^-transformed MDCK cells can escape from tumor suppressive polarized epithelium. (**a**) Confocal images of WT MDCK cells or MDCK cells transduced with HIF2α^2A^-, Enhancer of zeste homolog 2 (EZH2), β-catenin^4A^-, H-Ras^V12^ or K-Ras^V12^-expressing viral vectors and cultured for 6 days in 3D Matrigel matrix. Nuclei were counterstained with 2-(4-amidinophenyl)-1H -indole-6-carboxamidine (DAPI) (blue) and F-actin was visualized with Alexa488-phalloidin (green). (**b**) Quantification of the cyst phenotypes. Data are presented as the mean±s.d. and comes from three independent experiments (*n*=100 for each condition). Statistical significance was determined by two-tailed *t*-test. *P-values<0.05, **P-values<0.01 and ***P-values<0.001. (**c**) Schematic of the single-cell lentiviral infection experiment on polarized MDCK cysts. Representative images (**d**) and quantification (**e**) of clonal expansion of control (GFP only), β-catenin^4A^-, H-Ras^V12^- or K-Ras^V12^-transduced (GFP-positive) cells imaged 5 days after infection. (**f**) Snapshots of time-lapse imaging of control (GFP, green) cells or K-Ras^V12^-transformed (K-Ras^V12^, green) MDCK cells stably expressing H2B-mRFP to visualize nuclei. Imaging was started ~3 days after single-cell infection (indicated as 0 h). Data are presented as the mean±s.d. and comes from three independent experiments. Scale bars=50 μm (**a**, **d** and **f**).

Integrins are important ECM receptors that are critical for cancer cell proliferation and invasion.^1, 2, 3, 5, 12^ Although integrin mutations are rare in cancers and integrins do not directly transform cells, they are often required for oncogene-induced tumorigenesis and metastasis.^1, 3, 16^ However, the underlying molecular mechanisms remain uncertain. To address these mechanistic links, we first expressed different oncogenes in MDCK cells to assess their ability to transform polarized epithelial cells. Three-dimensional (3D) cultures of MDCK cells have been successfully used as a model to examine abnormal cell growth and polarity, both of which are features of tumorigenic cells.^5, 17^ Activating mutations or overexpression of HIF2α, Enhancer of zeste homolog 2, β-catenin, K-Ras and H-Ras are particularly frequent in solid tumors.^18, 19, 20, 21, 22^ Overexpression of β-catenin^4A^, H-Ras^V12^ or K-Ras^V12^ all led to severely compromised cyst formation resulting in cell clusters with poorly polarized outer epithelial layer surrounding a mass of non-polarized cells (Figures 1a and b). In contrast, HIF2α^2A^- and Enhancer of zeste homolog 2-overexpressing MDCK cells formed polarized cysts with single lumen with similar frequency as wild-type (WT) parental MDCK cells (Figures 1a and b).

Although on one hand, loss of epithelial polarity is a hallmark of aggressive tumors, on the other hand, polarized epithelial cells are remarkably resistant to transformation.^8, 9^ Therefore, epithelial organization *per se* has tumor-suppressing properties.^23^ Nevertheless, some oncogenes are capable of driving apical or basolateral extrusion of transformed cancer cells away from the tumor suppressing polarized epithelial monolayer (Supplementary Figure 1).^9, 24^ To focus our attention on strong oncogenes that can overcome the suppressive polarized epithelial microenvironment, we transformed individual cells in polarized MDCK cysts with β-catenin^4A^, K-Ras^V12^ or H-Ras^V12^ and analyzed their capacity to drive apical or basolateral cell extrusion or outgrowth.^9^ MDCK cells were allowed to form polarized cysts after which they were exposed to highly diluted lentivirus preparations expressing either green fluorescent protein (GFP) alone or both GFP and K-Ras^V12^, resulting in transduction of single cells within cysts^9^ (Figure 1c). Five days after, viral transduction cysts were fixed and the growth of GFP-positive cells was analyzed (Figures 1d and e). Long-term, time-lapse (48–60 h), live-cell spinning disc confocal microscopy was used to follow how the GFP-positive outgrowths formed (Figure 1f and Supplementary Movies 1 and 2). Neither outgrowth nor extrusion was observed for GFP-expressing cells (Figures 1d–f and Supplementary Movie 1). In contrast, K-Ras^V12^-overexpressing cells proliferated rapidly and formed bulging outgrowths within the epithelial layer and were frequently extruded from the cysts either basally or apically (Figures 1d–f and Supplementary Movie 2). In summary, K-Ras^V12^ and H-Ras^V12^ but not β-catenin^4A^ expression enabled outgrowth and extrusion of transformed cells, despite the surrounding normal epithelium. K-Ras^V12^ mutations are most frequently in many cancers.^18^ Therefore, we focused our studies on the effects of K-Ras^V12^ expression on integrin-mediated cell–ECM signaling, in order to reveal the escape mechanisms employed by the K-Ras^V12^-transformed cells.

### The expression of the components mediating cell–ECM interactions is modulated in K-Ras^V12^-transformed MDCK cells

**Figure 2:**
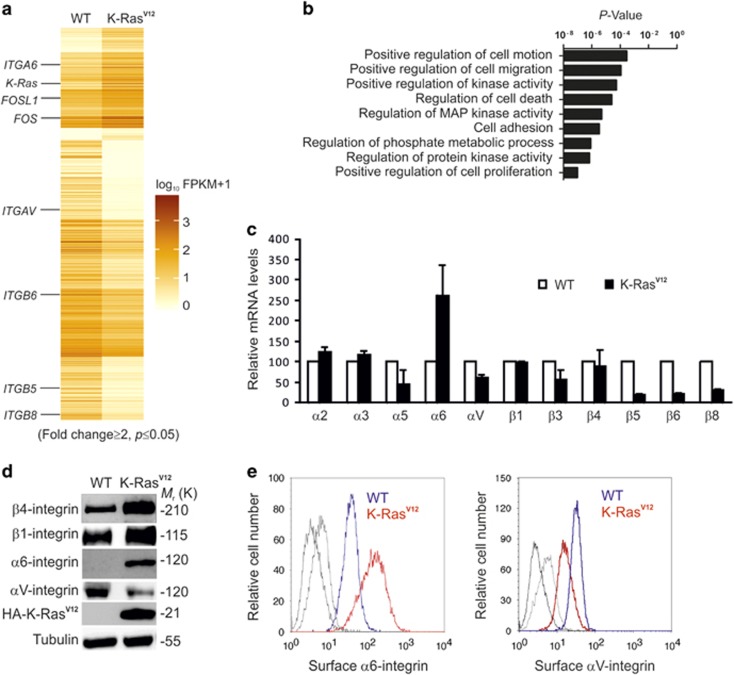
K-Ras^V12^ modulates integrin expression profile of transformed MDCK cells. (**a**) Heatmap representation of RNA-Seq analysis of WT- and K-Ras^V12^-MDCK cells showing differentially expressed transcripts (>2-fold; *P*<0.05). Deeper color shows higher expression level. FPKM, fragments per kilobase per million mapped fragments. (**b**) PANTHER pathway ontology analysis showing the most significantly affected biological processes modulated in K-Ras^V12^-expressing cells (*P*<0.05). (**c**) qPCR analysis of the relative expression levels of selected integrin subunits in WT and in K-Ras^V12^-expressing MDCK cells. (**d**) Western blot analysis was performed to detect β1-integrin, β4-integrin, α6-integrin, αV-integrin, HA (HA-K-RasV12) and tubulin in WT- and K-Ras^V12^-MDCK cells. (**e**) FACS analysis of surface expressed α6-integrin in WT (blue) and K-Ras^V12^ (red) MDCK cells or αV-integrin in WT (blue) and K-Ras^V12^ (red) MDCK cells. Unlabeled cells (only secondary antibody) are shown in gray.

To study the genetic program underlying phenotypic changes in K-Ras^V12^-transformed cells, the steady-state mRNA expression profile was analyzed by using RNA sequencing (RNA-Seq) for both WT- and K-Ras^V12^-MDCK cells (Figure 2a and Supplementary Table 1a). As expected, the PANTHER pathway ontology analysis revealed multiple K-Ras^V12^-induced changes in the MAP kinase pathway components and its downstream targets, which are involved in the regulation of cell proliferation and growth (Figure 2b). Importantly, cell adhesion and cell–ECM interaction-associated pathways, in which integrins are central nodes, were among the major pathways highlighted in the analysis (Figure 2b). *ITGA6* was highly upregulated, whereas αV-class integrin genes *ITGAV*, *ITGB5*, *ITGB6* and *ITGB8* were downregulated (Figure 2a). RNA-Seq data on integrins was confirmed using quantitative PCR (qPCR) (Figure 2c). Moreover, western blot analysis and fluorescence-activated protein kinase (FACS) cell surface analysis showed that the relatively low protein levels of α6-integrins in WT-MDCK cells are robustly upregulated upon expression of K-Ras^V12^ but αV-integrins are downregulated (Figures 2d and e). Previous studies have indicated that α6β4-integrins promote tumorigenesis but α6β1-integrins inhibits tumor progression.^25, 26, 27^ Whereas α6-integrin can pair with both β1- and β4-integrins to form heterodimeric laminin receptors,^28^ only α6β4-integrins were detected in co-immunoprecipitation assays in WT-MDCK and K-Ras^V12^-MDCK cells (Supplementary Figure 2a).

### α6-integrin expression is required for K-Ras^V12^-induced transformation and lung metastasis

**Figure 3:**
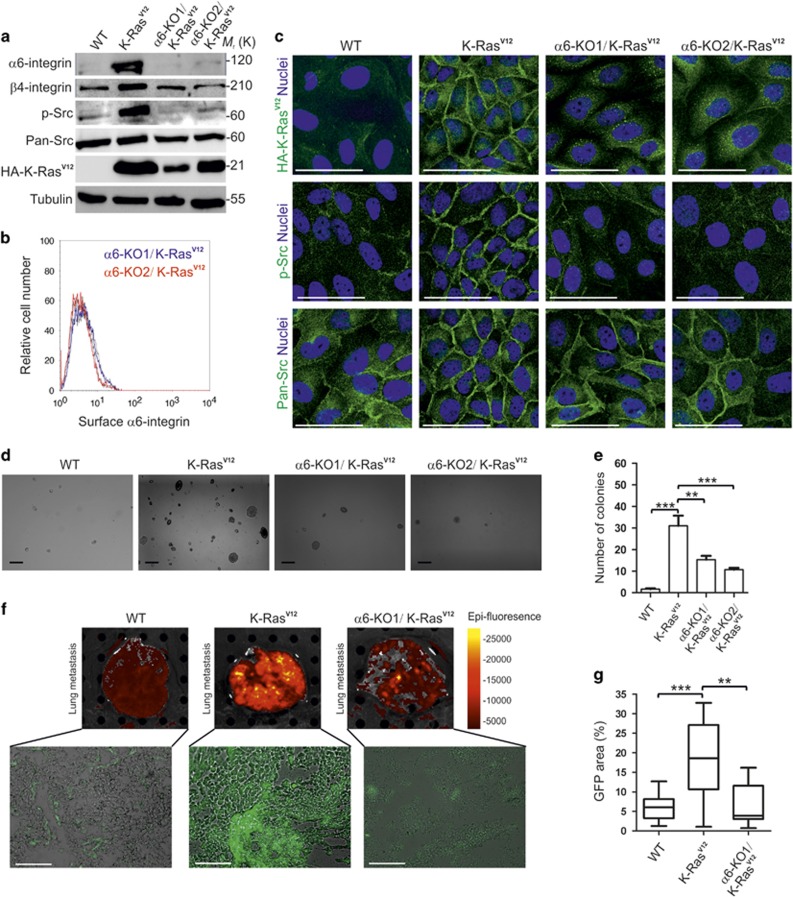
α6-integrin is required for K-Ras^V12^-induced anoikis resistance. (**a**) Western blot analysis of WT, K-Ras^V12^ and two independent α6-KO/K-Ras^V12-^MDCK cell line lysates using antibodies against α6-integrin, β4-integrin, phosphorylated-Src (pTyr^416^; p-Src), pan-Src, Hemagglutinin (HA)-tagged K-Ras^V12^ (HA-K-Ras^V12^) and tubulin. (**b**) FACS analysis of surface expressed α6-integrin in WT (left panel; blue) and K-Ras^V12^ (left panel; red) MDCK cells or α6-KO1/K-Ras^V12^ (right panel; blue) and α6-KO2/K-Ras^V12^ (right panel; red). Unlabeled cells (only secondary antibody) are shown in gray. (**c**) Confocal sections of WT-, K-Ras^V12^- and α6-KO/K-Ras^V12^-MDCK cells stained for K-Ras^V12^ (HA-K-Ras^V12^), Tyr^416^-phosphorylated-Src (p-Src) and total Src (pan-Src). Nuclei were visualized by 2-(4-amidinophenyl)-1H -indole-6-carboxamidine (DAPI). Scale bars=100 μm. (**d**) WT-, K-Ras^V12^, α6-KO1/K-Ras^V12^ and α6-KO2/K-Ras^V12^-MDCK cells were grown in soft agar and colony formation efficiency was determined as previous described.^68^ Scale bars=100 μm. (**e**) Quantification of colony formation in WT, K-Ras^V12^, α6-KO1/K-Ras^V12^ and α6-KO2/K-Ras^V12^ MDCK cells cultured in soft agar for 15 days. (**f**) Representative images of whole lungs from mice injected with GFP-expressing WT-, K-Ras^V12^ and α6-KO1/K-Ras^V12^-MDCK cells using IVIS Spectrum imaging system (upper panel). Representative fluorescence microscopy images of cryosections from lungs (lower panels). Scale bars=100 μm. (**g**) Quantification of lung metastases as area of GFP-positive colonies relative to the total lung tissue area. Data are presented as the mean±s.d. At least 10 randomly selected slides were analyzed per each sample. Statistical significance was determined using two-tailed *t*-test. *P-values<0.05, **P-values<0.01 and ***P-values<0.001.

To study whether α6-integrin function is necessary for K-Ras^V12^-induced transformation and metastasis, we generated α6-integrin knockout (α6-KO) MDCK cells using CRISPR/Cas9-technology followed by overexpression of K-Ras^V12^.^29^ Ablation of α6-integrin expression was confirmed by western blotting and FACS cell surface analysis (Figures 3a and b). To assess the transforming capacity of K-Ras^V12^ expression, cells were seeded into soft agar and grown for 15 days during which time WT-MDCK cells grew slowly and formed only small foci due to lack of proper ECM contact (Figure 3d). In contrast, K-Ras^V12^-MDCK cells formed multiple large cell clusters, indicating that they had acquired resistance to anoikis. K-Ras^V12^/α6-KO-MDCK cells had significantly reduced capacity to form colonies in soft agar when compared with K-Ras^V12^ cells (Figures 3d and e). The proto-oncogene c-Src (Src) is a non-receptor tyrosine kinase protein, which is associated with multiple aggressive cancers.^30, 31^ Recent studies have implicated Src tyrosine kinase as an important upstream activator of K-Ras-mediated signaling.^32, 33^ Integrin ligation is often associated with activation of a dual kinase complex involving focal adhesion kinase and Src.^34^ In order to examine whether Src-activation is affected by depletion of α6-integrin, we studied the levels of phosphorylated Src in the different cell lines. K-Ras^V12^ efficiently induced Src phosphorylation that was completely abolished upon depletion of α6 expression (Figure 3a). In addition, β4-integrin is also required for the activation of Src by K-Ras^V12^ (Supplementary Figure 2b). Strikingly, we observed that K-Ras^V12^ was redistributed from lateral plasma membrane domain to perinuclear vesicles in α6-KO cells (Figure 3c). Although Src tyrosine phosphorylation was strongly inhibited, the total levels or localization of Src protein remained unchanged (Figure 3c). To investigate *in vivo* metastatic capacity of WT-, K-Ras^V12^- and K-Ras^V12^/α6-KO-MDCK cells, they were injected into the tail veins of immunocompromised NOD.CB17-PrkdcSCID mice. All mice were killed 15 days later, except for three K-Ras^V12^-MDCK-injected mice that had to be killed earlier due to their poor condition. As all of the injected cells also expressed GFP, we measured the GFP fluorescence in the freshly dissected lung tissues using *in vivo* imaging system to evaluate the colonization of the lungs by the injected cells (Figure 3f). Then, part of the samples were prepared for cryosections and the remaining part for fixation for paraffin sections. Cryosections were imaged and the relative amounts of GFP-positive areas representing metastatic lesions were determined (Figures 3f and g). K-Ras^V12^-transformed MDCK cells had colonized significant parts of the lung tissue sections, whereas only occasional GFP-positive cells were observed in the lungs of mice injected with GFP-WT-MDCK cells (Figures 3f and g). Importantly, when compared with K-Ras^V12^-MDCK cells, the K-Ras^V12^/α6-KO-MDCK cells showed dramatically reduced lung metastasis (Figures 3f and g). Same results were seen when paraffin-embedded, hematoxylin and eosin-stained sections were analyzed for the size of tumor cell colonies (Supplementary Figure 6). Taken together, these data show that K-Ras^V12^-induced upregulation of α6-integrin expression is critical for oncogenic K-Ras^V12^ induced transformation and metastasis. Moreover, this function involves activation of Src kinase that in turn is required to maintain K-Ras^V12^ at the cell surface.

### Loss of α6-integrin inhibits anchorage-independent growth of K-Ras^V12^-transformed MDCK cells via suspension-induced apoptosis

**Figure 4:**
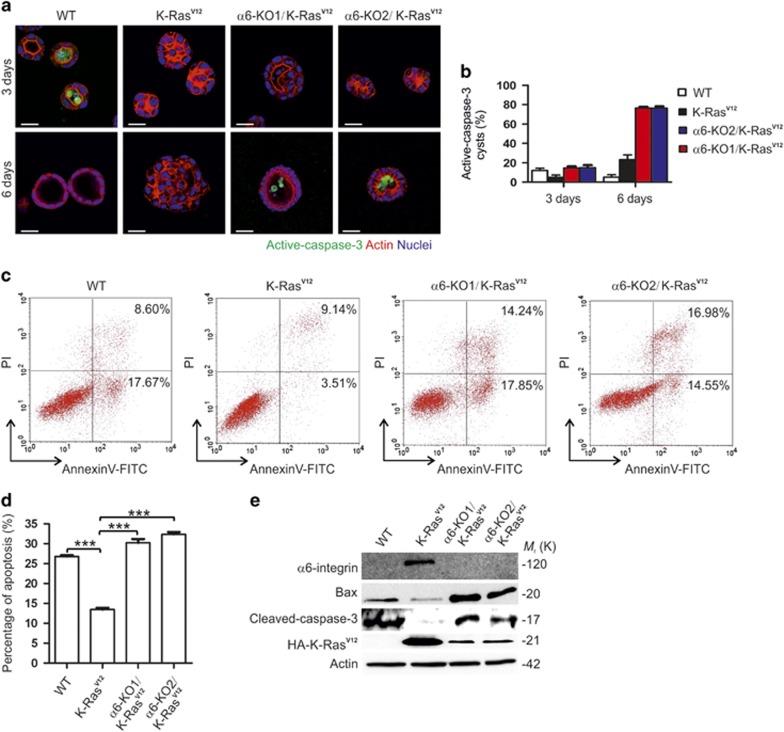
Knockout of α6-integrin promotes anoikis in K-Ras^V12^-MDCK cells. (**a**) Confocal sections of WT-, K-Ras^V12^-, α6-KO1/K-Ras^V12^- and α6-KO2/K-Ras^V12^-MDCK cells cultured for 3 (upper panel) or 6 days (lower panel) in 3D Matrigel matrix substrate and stained for active-caspase 3 (green). Nuclei were counterstained with 2-(4-amidinophenyl)-1H -indole-6-carboxamidine (DAPI) (blue) and F-actin was stained with TRITC-phalloidin (red). (**b**) Quantification of cleaved-caspase-3-positive cysts in WT-, K-Ras^V12^-, α6-KO1/K-Ras^V12^- and α6-KO2/K-Ras^V12^-MDCK 3D cultured cysts. (**c** and **d**) Anoikis in WT, K-Ras^V12^ cells and α6-KO/K-Ras^V12^ cells grown on polyHEMA-coated plates for 24 h was evaluated by Annexin-V/propidium iodide staining. Data are presented as the mean±s.d. and comes from three independent experiments. Statistical significance was determined using two-tailed *t*-test. *P-values<0.05, **P-values<0.01 and ***P-values<0.001. (**e**) Western blot analysis of pro-apoptotic protein Bax and apoptosis marker cleaved caspase-3 in WT, K-Ras^V12^ cells and α6-KO/ K-RasV12 cells grown on polyHEMA-coated plates for 24 h.

As stated above, reduction of α6-integrin decreased the number of K-Ras^V12^-MDCK cell colonies in soft agar assay and the metastatic colonization in the lungs. Whereas α6-integrin expression was not essential for basal outgrowth or extrusion of K-Ras^V12^-transformed cells within polarized cysts, apical extrusion was not observed (Supplementary Figure 3). To confirm and extend these results, we analyzed the growth of the different cell lines in 3D cultures in more detail. It was found that, similar to WT-MDCK cells, K-Ras^V12^/α6-KO-MDCK cells formed visible lumens when grown in 3D culture conditions, albeit with somewhat slower kinetics WT-MDCK cells (Figure 4a). To assess the role of anoikis in cystogenesis, the cysts were stained for active caspase-3 to label apoptotic cells. During the first 3 days, rare apoptotic cells were seen in the forming lumen of WT-MDCK cells (Figures 4a and b). Apoptotic cells were not detected in K-Ras^V12^-cell clusters but they were abundant in the lumens of K-Ras^V12^/α6-KO-MDCK cysts analyzed at day 6, indicating that luminal cells are cleared by apoptosis in the absence of α6-integrins (Figures 4a and b). To determine a role of α6-integrin in anoikis, we used flow cytometry to measure the percentage of apoptotic cells in WT-, K-Ras^V12^, K-Ras^V12^/α6-KO-MDCK cells seeded onto polyHEMA-coated plates. Loss of α6-integrin clearly increased the apoptotic cell population in K-Ras^V12^-expressing cells (Figures 4c and d). Western blot assay of cleaved caspase-3 from polyHEMA-grown cells further confirmed that loss of α6-integrin promoted anoikis-induced cell death in K-Ras^V12^-expressing cells. Furthermore, we found that K-Ras^V12^-mediated inhibition of pro-apoptotic Bax expression was reversed upon α6-KO in polyHEMA-grown cells (Figure 4e).

### K-Ras^V12^ upregulates α6-integrin expression via MAPK-mediated activation of FOSL1

**Figure 5:**
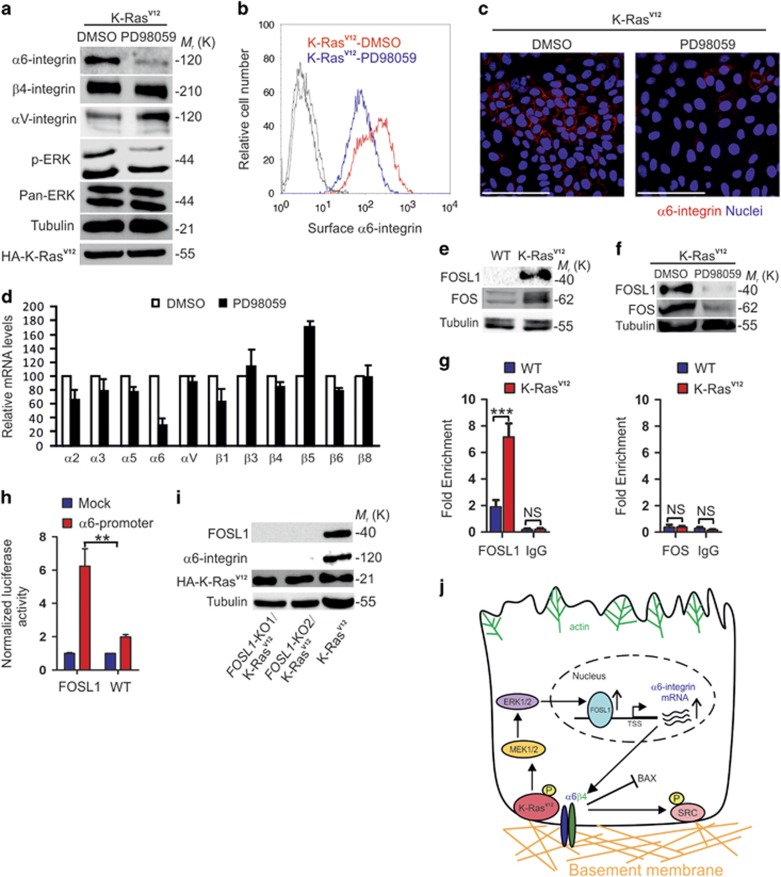
K-Ras^V12^ upregulates α6-integrin expression via MAPK/ERK/FOSL1 pathway. (**a**) MDCK cells were transduced with HA-K-Ras^V12^, cultured for 8 days with dimethyl sulfoxide (DMSO) (0.05%) or PD98059 (10 μM in 0.05% DMSO) and subjected to immunoblotting with antibodies against α6-integrin, β4-integrin, phospho-ERK1/2 (p-ERK), total ERK (Pan-ERK), HA (HA-K-Ras^V12^) or tubulin. (**b**) FACS analysis of the surface expressed α6-integrin in mock (DMSO; red) or PD98059 (10 μM; blue)-treated K-Ras^V12-^MDCK cells. (**c**) Confocal section of mock (DMSO) and PD98059 (10 μM)-treated K-Ras^V12^ MDCK cells stained for α6-integrin (red). Nuclei were counterstained with 2-(4-amidinophenyl)-1H -indole-6-carboxamidine (DAPI) (blue). Scale bars=100 μm. (**d**) Relative mRNA expression levels of selected integrin subunits in mock (DMSO, white) and PD98059 (10 μM; black)-treated K-Ras^V12-^MDCK cells. (**e**) Immunoblotting of FOSL1 and FOS in WT- and K-Ras^V12^-MDCK cells. Tubulin antibody was used as a loading control. (**f**) Mock (DMSO)- or PD98059 (10 μM)-treated K-Ras^V12^-MDCK cells were subjected to immunoblotting as described in (**f**). (**g**) WT and K-Ras^V12-^MDCK cells were subjected for chromatin immunoprecipitation (ChIP)-qPCR analysis using either FOSL1- (left panel) or FOS- (right panel) antibodies and primers amplifying selected regions at the α6-integrin promoter. Data are presented as the mean±s.d. and come from three independent experiments. Statistical significance was determined using two-tailed *t*-test. *P-values<0.05, **P-values<0.01 and ***P-values<0.001. (**h**) Luciferase reporter assays showing increased transcriptional activity of α6-integrin promoter upon ectopic expression of FOSL1 in MDCK cells. Data are presented as the mean±s.d. and come from three independent experiments. Statistical significance was determined using two-tailed *t*-test. *P-values<0.05, **P-values<0.01 and ***P-values<0.001. (**i**) Western blot analysis of K-Ras^V12-^and FOSL1-KO/K-Ras^V12^-MDCK cells with antibodies against α6-integrin, FOSL1, HA (HA-K-Ras^V12^) and tubulin. (**j**) Schematic model of K-Ras^V12^-mediated upregulation of α6-integrin expression through activation of the MAP/ERK/FOSL1-cascade to stimulate α6-integrin transcription and inhibit Bax expression. α6-integrin-dependent signaling crosstalk between K-Ras^V12^ and Src is also indicated.

Next we investigated the mechanism how α6-integrin is upregulated by K-Ras^V12^. We tested small molecule inhibitors targeting ERK (PD980259) and PI3K (Pictilisib), the two major kinase cascades activated by the Ras pathway.^35, 36^ It was observed that PD980259 efficiently blocked the K-Ras^V12^-induced expression of α6-integrin (Figures 5a–c), whereas Pictilisib was without an effect (Supplementary Figure 4). PD980259 downregulated α6-integrin expression at both protein and mRNA level, suggesting a transcriptional control mechanism (Figures 5a–d). To explore the possible mechanisms of transcriptional regulation of α6-integrin expression, we analyzed the RNA-seq data focusing on transcription factors. MAPK/ERK pathway effectors, *FOS* and *FOSL1*, were significantly upregulated in K-Ras^V12^-transformed MDCK cells (Figure 2a and Supplementary Table 1a).^37^ Upregulation of the gene products of *FOS* and *FOSL1* was confirmed by western blotting (Figure 5e). Treatment of K-Ras^V12^-MDCK cells with the ERK inhibitor, PD980259, prevented upregulation of both FOS and FOSL1 (Figure 5f). The promoter region of *ITGA6* contain potential binding sites for FOS and FOSL1. To study whether FOS or FOSL1 bind to the *ITGA6* promoter region, we performed chromatin immunoprecipitation assay followed by qPCR in WT- and K-Ras^V12-^MDCK cells. We found that in K-Ras^V12^-MDCK cells, only FOSL1 was enriched at the *ITGA6* promoter region (Figure 5g). Furthermore, our luciferase reporter assay demonstrated that *ITGA6* is a direct target of FOSL1 in MDCK cells (Figure 5h). To confirm a functional role for FOSL1, we used CRISPR/Cas9 to knockout FOSL1 expression in K-Ras^V12^-MDCK cells. In agreement with the important role of FOSL1 in inducing *ITGA6* transcription, α6-integrin expression was not seen in K-Ras^V12^/FOSL1-KO-MDCK cells (Figure 5i). These data show that K-Ras^V12^ activates the MAPK/ERK pathway leading to FOSL1 binding to *ITGA6* promoter, thereby inducing transcription of *ITGA6* (Figure 5j). Importantly, all the functional phenotypes observed for K-Ras^V12^/α6-KO MDCK cells, such as the loss of anoikis resistance, inhibition of proapoptotic Bax expression and reduced lung colonization when compared with K-Ras^V12^ MDCK cells, could be replicated using K-Ras^V12^/FOSL1-KO cells (Supplementary Figures 5 and 6).

### Restoration of αV-integrin expression in K-Ras^V12^-transformed cells results in EMT

**Figure 6:**
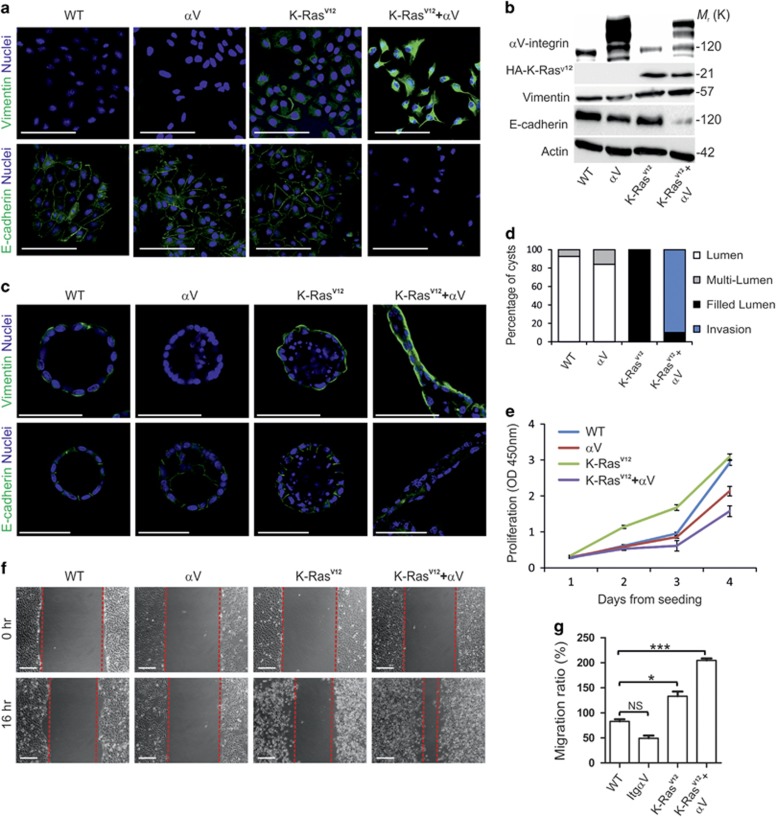
K-Ras^V12^ and αV-integrins synergize to induce EMT in MDCK cells (**a**) Confocal section of WT-, αV-RFP-, K-Ras^V12^- and K-Ras^V12^/αV-RFP-MDCK cells stained for vimentin (green; upper panels), E-cadherin (green; lower panels). Nuclei were counterstained with DAPI (blue). Scale bars, 100 μm. (**b**) Immunoblotting of WT-, αV-RFP-, K-Ras^V12^- and K-Ras^V12^/αV-RFP-MDCK cells with antibodies against αV-integrin, HA (HA-K-Ras^V12^), vimentin, E-cadherin and actin. (**c**) Confocal sections of WT-, αV-RFP-, K-Ras^V12^- and K-Ras^V12^/αV MDCK cells grown as 3D cyst cultures are shown. For confocal microscopy cysts were stained for vimentin (green; upper panels) or E-cadherin (green; lower panels). (**d**) Quantification of lumen formation in the 3D cultures. Data is combined from at least three independent experiments (*n*~100 for each condition). Scale bar, 50 μm. Data are presented as the mean±s.d. and comes from three independent experiments. (**e**) Cell proliferation was measured at the indicated time points by XTT colorimetric assay (absorbance at 450 nm (OD 450); mean±s.d. of five independent experiments). (**f**) Wound-healing assay of WT, αV-RFP-, K-Ras^V12^- and K-Ras^V12^/αV-RFP-MDCK cells. (**g**) Relative rates of cell migration was determined from three independent wound healing experiments. Statistical significance was assessed using two-tailed *t*-test. *P*-values <0.05 are signified by (*), <0.01 by (**) and <0.001 by (***). Scale bar, 150 μm. Data are presented as the mean±s.d. and comes from three independent experiments.

Several subunits belonging to the αV-class integrins were downregulated in K-Ras^V12^ MDCK cells (Figures 2c–e). αV-integrins have been implicated in cellular mechanotransduction and they are known to bind to and activate latent TGF-β1, thereby contributing to EMT, presumably via activation of Src/focal adhesion kinase pathway.^38^ In light of these data, downregulation of αV-class integrins K-Ras^V12^-cells seems counterintuitive. However, this could be a cell intrinsic regulatory mechanism in non-tumorigenic MDCK cells to restrict cell transformation. Moreover, loss of epithelial polarity and integrity *in vivo* is often linked with tumor-associated inflammation and fibrosis that in turn is known to induce αV-integrin expression in epithelial cells.^38, 39^ In order to clarify the possible role of αV-class integrins in K-Ras^V12^-driven transformation, we utilized an ectopic cytomegalovirus-based promoter to overexpress αV-integrin fused to red fluorescent protein (αV-RFP) in both WT- and K-Ras^V12^-MDCK cells. The MDCK cells expressing both K-Ras^V12^ and αV-integrins displayed striking phenotypic differences when compared with other cell lines, as they appeared to loose extensive E-cadherin-positive cell–cell contacts and acquired an elongated mesenchymal morphology (Figure 6a). Immunofluorescence and western blot studies confirmed downregulation of E-cadherin and upregulation of vimentin, suggesting that overexpression of K-Ras^V12^ and αV-RFP synergistically induced EMT in MDCK cells (Figures 6a and b). The invasive properties of K-Ras^V12^/αV-RFP-MDCK cells were especially evident when they were grown in 3D Matrigel cultures, where they migrated to form interconnected cellular networks without obvious polarity or lumen (Figures 6c and d). Whereas K-Ras^V12^-MDCK cells proliferated with accelerated kinetics, the growth rate of K-Ras^V12^/αV-RFP-MDCK cells was significantly slower when compared with WT-MDCK cells or cells expressing only αV-RFP (Figure 6e). Moreover, wound-healing assay revealed that αV-RFP and K-Ras^V12^ synergistically induced cell migration (Figures 6f and g). In the single-cell transformation assay, it was found that basal outgrowth was clearly favored over apical extrusion by αV-RFP-expressing K-Ras^V12^ cells (Supplementary Figure 7).

### K-Ras^V12^ synergize with αV-integrins to induce EMT via activation of TGF-β pathway

**Figure 7:**
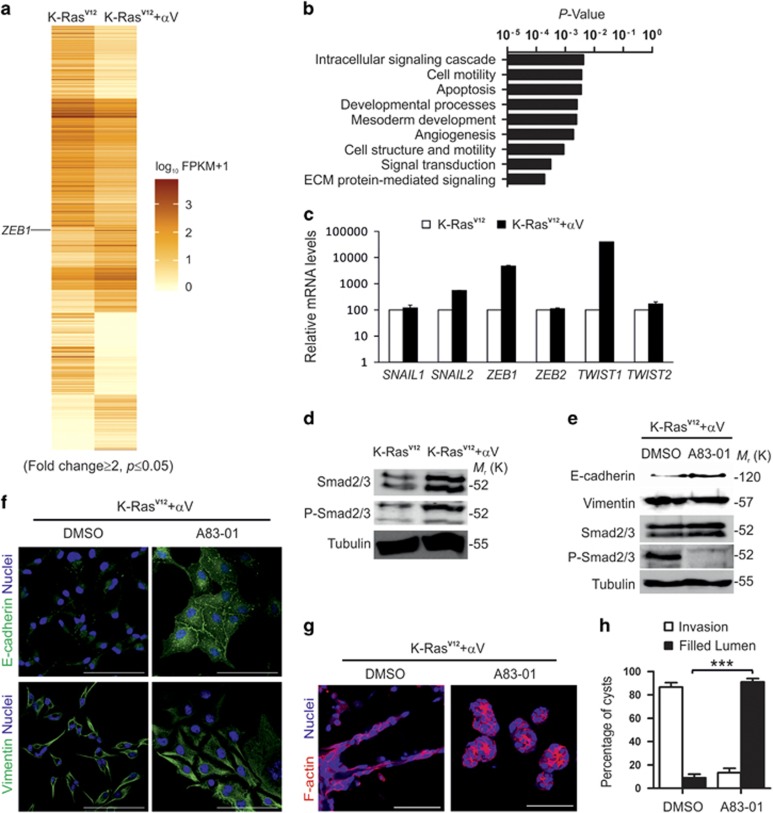
K-Ras^V12^ and αV-integrins induce EMT via activation of TGF-β pathway in MDCK cells. (**a**) Heat-map representation of RNA-Seq analysis of K-Ras^V12^- and K-Ras^V12^-expressing MDCK cells showing differentially expressed transcripts (>2-fold; *P*<0.05). Deeper color indicates higher expression level. FPKM, fragments per kilo-base per million mapped fragments. (**b**) PANTHER pathway ontology analysis showing the most significantly affected biological processes modulated by αV-RFP expression in K-Ras^V12^-MDCK cells (*P*<0.05). (**c**) qPCR analysis of EMT-related TF mRNA expression levels in K-Ras^V12^ and K-Ras^V12^/α-RFP-MDCK cells. (**d**) Western blot analysis was performed to detect Smad2/3, Phospho-Smad2/3 and tubulin in K-Ras^V12^ and K-Ras^V12^/αV-RFP-MDCK cells. (**e**) Western blot analysis was performed to detect E-cadherin, vimentin, Smad2/3, Phospho-Smad2/3 and tubulin in K-Ras^V12^/αV-RFP-MDCK cells treated with dimethyl sulfoxide (DMSO) or TGF-β inhibitor A83-01. (**f**) Confocal section of K-Ras^V12^/αV-RFP-MDCK cells treated with DMSO or TGF-β inhibitor A83-01 and stained for E-cadherin (green; upper panels) or vimentin (green; lower panels). Nuclei were counterstained with 2-(4-amidinophenyl)-1H -indole-6-carboxamidine (DAPI) (blue). Scale bars=50 μm. (**g**) Confocal sections (left and right panels) of 3D cyst cultures of K-Ras^V12^/αV MDCK cells treated with DMSO or TGF-β inhibitor A83-0. Cysts were stained for F-actin (red) and nuclei were counterstained with DAPI (blue). Scale bars=50 μm. (**h**) Quantification of cyst phenotypes in the 3D cultures from **g**. Data are combined from at least three independent experiments (*n*~100 for each condition). Scale bar=50 μm. Data are presented as the mean±s.d.

To study in more detail the transcriptional program triggered in K-Ras^V12^/αV-RFP-MDCK cells an RNA-seq based transcriptomic analysis was performed comparing K-Ras^V12^ cells expressing or not αV-RFP (Figure 7a and Supplementary Table 1b). PANTHER analysis highlighted ECM-mediated signaling as the most significantly modified pathway (Figure 7b). Given the observed EMT in K-Ras^V12^/αV-RFP cells, we focused our analysis on the known EMT-inducing transcription factor genes *SNAI1*, *SNAI2*, *ZEB1*, *ZEB2*, *TWIST1* and *TWIST2*, all of which have been implicated in cancer cell metastasis.^40^ Although RNA-Seq analysis identified only *ZEB1* as a gene highly upregulated by K-Ras^V12^/αV-RFP co-expression, a qPCR analysis detected more than 395-fold upregulation of *TWIST1* and 5.5-fold upregulation of *SNAI2* (Figure 7c). Taken together, these data show that αV-integrins cooperate with K-Ras^V12^ to drive EMT likely through upregulation of *TWIST1*, *ZEB1* and *SNAI2* expression. K-Ras^V12^ expression alone rendered MDCK cells anoikis-resistant but failed to induce EMT due to efficient downregulation of αV-class integrins. Previous studies show that αV-integrins modulate the activation of TGF-β signaling, such as active latent TGF-β.^41^ We could show induced Smad2/3 phosphorylation in αV-RFP-expressing K-Ras^V12^-MDCK cells (Figure 7d). Next, we used a TGF-β inhibitor (A83-01) to inhibit TGF-β-signaling in K-Ras^V12^/αV-RFP-MDCK cells (Figure 7e). Interestingly, A83-01 treatment not only inhibited Smad-phosphorylation but it also upregulated E-cadherin expression and led to formation of extensive E-cadherin-positive adherens junctions (Figures 7e and f). Remarkably, the invasive behavior of K-Ras^V12^/αV-RFP-MDCK cells in 3D Matrigel was essentially blocked (Figures 7g and h). Therefore, we concluded that re-expression of αV-integrins in K-Ras^V12^-MDCK cells led to autocrine activation of TGF-β signaling resulting in EMT.

### α6-integrin, FOSL1 and αV-integrins are co-expressed in aggressive human cancers harboring activating K-Ras mutations

**Figure 8:**
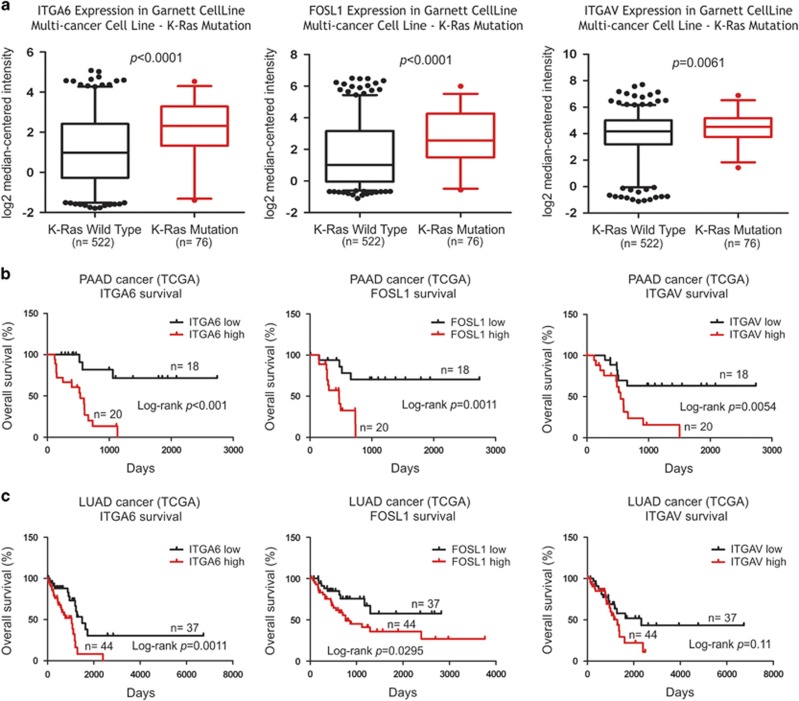
High levels of α6-integrin, FOSL1 or αV-integrin expression correlate with aggressive phenotypes in mutant K-Ras-driven human cancers. (**a**) ITGA6, FOSL1 and ITGAV mRNA expression was determined from cancer cell lines with either WT K-Ras (*n*=522) or with K-Ras mutation (*n*=76) from the Garnett *et al.*^42^ cancer cell line data set. Gene expression intensity is shown as the log2 median-centered intensity, as reported in Oncomine.^69^ (**b**) Kaplan–Meier plots showing the survival of pancreatic adenocarcinoma patients (PAAD) and (**c**) lung adenocarcinoma (LUAD) patients with either high (top 10% highest expression; *n*=20 for PAAD; *n*=44 for LUAD) or low (bottom 10% lowest expression; *n*=18 for PAAD; *n*=37 for LUAD) expression of ITGA6, FOSL1 and ITGAV. (queried from TCGAbrowser^45^).

To assess whether high expression levels of α6-integrin and FOSL1 may underlie K-Ras-driven human cancer progression, we performed an analysis of human cancer cell lines whose K-Ras mutation status has been documented. Gene expression analysis was done by combining data from 522 cancer cell lines with WT K-Ras and 76 cell lines with mutated K-Ras.^42^ Notably, we observed robustly elevated levels of both α6-integrin and FOSL1 expression in K-Ras mutant human cancer cell lines (Figure 8a). Similarly, αV-expression was significantly upregulated in mutant K-Ras-expressing cell lines. To analyze the potential relevance of high expression levels of these genes for cancer progression in patients, we went on to analyze publicly available clinical cancer data sets. Analysis of The Cancer Genome Atlas data sets using cBioportal platform for cross-cancer alteration and expression analysis for K-Ras revealed that, among the 20 types of cancers in the database, pancreatic cancer and lung cancer had clearly the highest rate of sequenced K-Ras mutations and expressed K-Ras at high level (Supplementary Figure 8).^43, 44^ Therefore, pancreatic adenocarcinomas and lung adenocarcinomas were selected for further analysis to explore whether the expression levels of α6-integrin, FOSL1 and αV-integrin were correlated with cancer progression and aggressiveness. The clinical relevance of α6-integrin, FOSL1 and αV-integrin upregulation was visualized by using Kaplan–Meier analysis with clinical survival data from a collection of 179 pancreatic and 516 lung cancer (adenocarcinoma) samples from TCGAbrowser,^45^ respectively. Strikingly, high levels of α6-integrin, FOSL1 and αV-integrin expression were all correlated with significantly reduced survival of both in pancreatic cancer and lung cancer patients when compared with patients with low level expression of the same genes (Figure 8).

## Discussion

Here we report that K-Ras^V12^-mediated transformation can overcome the suppressive effect of polarized epithelial microenvironment leading to basal outgrowth or apical cell extrusion. Survival of extruded cells without proper ECM contact depended on α6-integrins whose expression was upregulated in K-Ras^12V^-transformed cells through activation of MAPK/ERK/FOSL1 pathway. High levels of α6-integrins have been found to be expressed in adult stem cells and in cancer stem cells.^46, 47, 48^ α6-integrin expression correlates with enhanced proliferation capacity, spheroid formation and, in the case of cancer stem cells, tumor initiation upon xenografting.^49, 50^ These data are in agreement with the K-Ras^V12^-induced α6-integrin-dependent anoikis resistance and increased metastatic potential observed in our current study.

Ras/MAPK/ERK pathway is important for growth of many types of cancer cells and it has been implicated in stemness signaling.^51^ Here we show that K-Ras/MAPK/ERK signaling cascade induces FOSL1 expression that in turn binds to α6-integrin promoter to stimulate transcription of α6-integrin. FOSL1 is also highly expressed in many epithelial cancers but its role during tumorigenesis is less clear, except that FOSL1 is thought to contribute to the regulation of EMT-associated transcription factors.^52^ Importantly, Ras signaling and EMT-inducing TGF-β signaling have been shown to collectively regulate epithelial cell plasticity and invasive migration.^53^ Our data not only corroborate these findings but we also demonstrate that two distinct integrins are essential mediators of this signaling interplay. We show here that although K-Ras^V12^-induced expression of α6-integrin promoted anchorage-independent growth, the ability of K-Ras^V12^ to trigger invasive behavior and EMT depended on αV-integrin expression, which upregulated expression of *TWIST1*, *ZEB1* and *SNAI2*. Altered αV-integrin functions have been observed in many types of cancer and in most cases αV-integrins modulate the activation of growth factor receptor signaling, including TGF-β.^54^ In particular, αV-integrins are directly involved in the activation of large latent TGF-β complex.^55^ Curiously, we observed downregulation of αV-integrins in K-Ras^V12^-transformed MDCK cells. In contrast, most cancer cells display elevated αV-integrin expression levels that also correlated with poor prognosis in mutant K-Ras-driven adenocarcinomas (Figure 8). The observed αV-integrin downregulation might represent a cell intrinsic mechanism, still intact in MDCK cells, which limits uncontrolled proliferation driven by constitutive K-Ras signaling. Inhibition of MAPK/ERK pathway partially restored αV-integrin expression in K-Ras^V12^-MDCK cells (Figure 5a). FOSL1, as part of SP1 transcription factor complex, has been reported to inhibit transcription of *ITGAV* and *ITGB3* in normal endothelial cells.^56^ However, the mRNA levels of αV-integrins were not affected in K-Ras^V12^-MDCK cells, suggesting an indirect regulatory mechanism that regulates the stability of αV-integrins at protein level. The molecular machinery by which K-Ras^V12^ inhibits αV-integrin levels is likely to be complex but it definitely warrants future research. The important finding in the current study is that re-expression of αV-integrins is sufficient to induce EMT in K-Ras^V12^-transformed cancer cells and might thus represent a second key mutagenesis hit that converts TGF-β from tumor suppressor to tumor-promoting factor.^57^

In conclusion, our data clarifies the molecular mechanisms by which K-Ras^V12^ mediates cell transformation and identifies FOSL1, ZEB1 and TWIST1 as crucial transcription factors regulated by oncogenic K-Ras^V12^. Importantly, our data shows that efficient K-Ras^V12^-mediated cell transformation depends on balanced activation of both α6- and αV-integrins, which promote anoikis resistance and invasive phenotype, respectively, to drive metastasis and aggressive tumorigenesis with poor prognosis. These findings may allow development of novel strategies to interfere with oncogenic properties of K-Ras^V12^ and thereby tackle mutant K-Ras-driven cancers that are generally difficult to treat.

## Materials and methods

### Cell culture and viral transduction

MDCK II cells (Heidelberg strain) were a kind gift from Dr K Simons (MPI-CBG, Dresden, Germany). Cells were grown in MEM media (Life Technologies, Thermo Fisher Scientific, Helsinki, Finland) supplied with 5% fetal bovine serum (Life Technologies, Thermo Fisher Scientific) and 1% penicillin/streptomycin (Life Technologies, Thermo Fisher Scientific), as described previously.^17^ Viral supernatants were generated by transfecting human embryonic kidney 293T cells (ATCC, LGC Standards GmbH, Wesel, Germany; CRL-11268) with the lentiviral transfer plasmids in combination with second-generation lentivirus packaging system^58^ using Lipofectamine 2000 (Invitrogen, Thermo Fisher Scientific). For ectopic expression, we constructed a pLVET-IRES-GFP vector from pLVET-tTR-KRAB (11644, Addgene, Cambridge, MA, USA)^59^ by replacing GFP-ttR-KRAB cassette with an IRES-GFP cassette from pMSCV-PIG (21654, Addgene).^60^ cDNAs of β-catenin^4A^, K-Ras^V12^ and H-Ras^V12^ were amplified by PCR from, E[beta]C (Addgene plasmid 24312),^21^ MSCV-H-RasV12-IRES-GFP (Addgene plasmid 18780) and pLenti-PGK-KRAS4B(V12) (Addgene plasmid 35633),^61^ respectively, and subsequently cloned using *Pme*I/*Bam*HI/*Eco*RI sites in pLVET-IRES-GFP vector. HA-HIF2α-P405A/P531A-pBabe-Hygro was a gift from William Kaelin (Addgene plasmid 21676).^22^ MSCV-hygro-F-Ezh2 (Addgene plasmid 24926)^62^ and pHIV-H2B-mRFP (Addgene plasmid 18982)^63^ were obtained from Addgene. cDNA of αV-integrin was amplified by PCR from pTag RFP-αV-integrin (EVROGEN, BioCat GmbH, Heidelberg, Germany) and cloned into RVH1-puro^64^ vector using *Xba*I and *Not*I. The cloning primers are listed in Supplementary Table 3. For CRISPR/Cas9 knockout, lentiCRISPR-v2 (Addgene plasmid 52961)^29^ was used. LentiCRISPR/α6-KO and lentiCRISPR/ FOSL1-KO plasmids were constructed as described previously.^29^ The DNA oligonucleotides used for gRNAs are listed in Supplementary Table 4.

### Antibodies and reagents

All primary antibodies used are listed in Supplementary Table 2. Cy3- and horseradish peroxidase-conjugated secondary antibodies were all from Jackson Immunochemicals (LABNET OY, Helsinki, Finland) and Alexa488-conjugated secondary antibodies were from Invitrogen. TRITC-Phalloidin and 2-(4-amidinophenyl)-1H -indole-6-carboxamidine were purchased from Sigma-Aldrich (Merck, Helsinki, Finland). Hygromycin B and Puromycin were from Thermo Fisher Scientific (Helsinki, Finland). dimethyl sulfoxide was from Sigma-Aldrich. The MEK inhibitor PD98059 was from LC Laboratories (Woburn, MA, USA). The PI (3) K inhibitor Pictilisib was from Selleck Chemicals (SMS Gruppen, Rungsted, Denmark).

### Three-dimensional cell culture

Three-dimensional overlay cultures were carried out as previously described.^9^ Briefly, 150 μl of Matrigel matrix (354230, BD Biosciences, Becton Dickinson Oy, Vantaa, Finland) was allowed to gel at 37 °C on a 35 mm glass-bottom μ-Dish (81158, IBIDI, Mediq Suomi Oy Laboratory Products, Espoo, Finland) suitable for fluorescence microscopy. Cells were seeded at ~5000 cells per dish in medium containing 2% (v/v) Matrigel matrix. Medium was replaced every 2 days with fresh medium containing 2% Matrigel matrix. For analysis, random fields were examined such that a minimum of 100 cysts per each condition were scored.

### Immunofluorescence

For immunofluorescence analysis, cells were fixed in PBS-3% paraformaldehyde for 20 min at room temperature, permeabilized with PBS-0.2% Triton X-100 and quenched with PBS-100 mM glycine, and then blocked with IF buffer (130 mM NaCl, 7 mM Na_2_HPO_4_, 3.5 mM NaH_2_PO_4_, 7.7 mM NaN_3_, 0.1% bovine serum albumin, 0.2% Triton X-100 and 0.05% Tween-20) for 50 min at room temperature. Samples were incubated with primary antibodies (see Supplementary Table 1 for details) in IF buffer overnight at 4 °C, rinsed three times with IF buffer at room temperature with gentle rocking, incubated with fluorescent-conjugated secondary antibody in IF buffer for 1 h at room temperature, washed three times with PBS, incubated 15 min with PBS containing 2-(4-amidinophenyl)-1H -indole-6-carboxamidine (Sigma-Aldrich), washed once with PBS and then mounted with ImmuMount (Thermo Scientific). Confocal laser scanning microscopy images were acquired by using a Zeiss LSM 780 confocal microscope, × 40 Plan-Apochromat objective (numerical aperture=1.4). Stacks of 1.5 μm were acquired with 100 nm increments from the basal region of cells containing matrix adhesions and analyzed with the ZEN 2011 software.

### Live-cell imaging

MDCK cells stably expressing H2B-mRed were set up for 3D culture in 35 mm glass-bottom μ-Dish (81158, IBIDI). Live imaging was performed with a custom-built spinning disk confocal system based on Zeiss spinning disc confocal microscopy with a spinning disc unit (Yokogawa CSU-X1), Hamamatsu electron-multiplying charge-coupled device camera and humidified, environmental control incubator (37 °C, 5% CO_2_). Images were acquired starting ~36 h after virus infection. Image stacks of complete cysts were captured with a EC Plan NeoFluar × 40/0.75 DIC (air) objective every 30 min for a duration of 48 h using Zen 2012 (Carl Zeiss Oy, Vantaa, Finland; Blue edition) software and were analyzed with Zen 2012 (Blue edition).

### RNA-seq and data analysis

Total RNA was extracted from the cells using Trizol Reagent (15596026, Life Technologies, Thermo Fisher Scientific). The concentration and quality of RNA samples were determined using a Nano Drop 2000 micro-volume spectrophotometer (Thermo Scientific). The library construction and sequencing were performed by the Beijing Genomics Institute-Hongkong, China (http://www.bgitechsolutions.com/), as described previously.^65^ Paired-end, 100 bp read-length sequencing was carried out using Illumina HiSeq 2000 and 40 million reads were generated for each sample. The RNA-seq reads were mapped to dog genome reference sequence (Broad CanFam3.1) by using TopHat2. Cuffdiff was used for differential gene expression analysis of RNA-seq data. Heatmap was generated by CummeRbund in R, as described previously.^66^ RNA-seq data are deposited at BioProject under accession number PRJNA382611.

### Western blotting

Cells were lysed on ice in RIPA lysis buffer (50 mM Tris/HCl, pH 8.0, 250 mM NaCl, 1% TritonX-100, 0.5% (w/v) sodium deoxycholate and 1 mM EDTA) containing complete mini-protease inhibitor cocktail (Roche, Roche Diagnostics Oy, Espoo, Finland). Lysates were sonicated and centrifuged at 13 000 r.p.m. for 10 min, 4 °C. Protein concentrations were determined with the BCA Protein Assay Kit (Pierce, Thermo Fisher Scientific). Proteins were separated using SDS–polyacrylamide gel electrophoresis (MiniProtean3 system, Bio-rad Laboratories, Helsinki, Finland) and transferred on Protran nitrocellulose filters (Perkin-Elmer, Turku, Finland) and then exposed to primary antibodies as described earlier.^17^ Horseradish peroxidase-conjugated secondary antibodies were visualized using a chemiluminescence kit (Pierce, Thermo Fisher Scientific) and LAS-3000 imaging system (Fuji-film, Fuji Finland, Vantaa, Helsinki). Uncropped original western blot full scans show in Supplementary Figure 9.

### FACS analysis

For surface FACS, cells were incubated with rat anti-a6-integrin (555841, BD Biosciences) in FACS buffer (0.5% bovine serum albumin in PBS) for 30 min at 4 °C and then washed twice in FACS Buffer. Cells were incubated with Alexa488-conjugated anti-mouse secondary antibodies (A-11001, Invitrogen) for 15 min and then washed twice in FACS Buffer for 30 min each at 4 °C. At least 10,000 cells were analyzed on a BD FACSCalibur cell analyzer (BD Biosciences) and FACS data were computed using the CellQuest software (Becton Dickinson Oy, Vantaa, Finland).

### Soft agar assays

About 2000 WT-, K-Ras^V12^-, α6-KO1/ K-Ras^V12^- or α6-KO2/ K-Ras^V12^-MDCK cells were suspended in 1 ml of prewarmed 0.35% SeaPlaque agarose/MEM/5% fetal bovine serum and pipetted on top of 1.5 ml of 0.7% SeaPlaque agarose/MEM/5% fetal bovine serum gelled onto a well of a six-well plate. Cells were incubated for 15 days, at 37 °C and 5% CO_2_, and fresh medium was exchanged twice per week. All colonies formed in each of the wells were imaged using an Olympus CellSens live-cell/time-lapse imaging system at room temperature. ImageJ were used to segment the images and count colonies that were larger than 100 μm in diameter.

### Chromatin immunoprecipitation and qPCR

ChIP assays were carried out as previously described.^58^ Cells were cross-linked in 1 % formaldehyde at room temperature for 10 min. The reaction was terminated with 125 mM glycine solution for 5 min followed by cell lysis and sonication (Q800R sonicator, QSonica, LLC., Newtown, CT, USA) to generate chromatin fragments with an average size of 0.3 kb. FOSL1 (PCRP-FOSL1-1E3, Developmental Studies Hybridoma Bank, Iowa City, IA, USA), FOS (sc-7202X, Santa Cruz Biotechnology, Thermo Fisher Scientific, Vantaa, Finland) and control IgG (normal mouse IgG and normal rabbit IgG, Jackson Immunochemicals) were used for the ChIP assay. ChIP DNA was purified with the Mini-Elute PCR purification kit (28006, Qiagen, Evo, Finland), and analyzed by quantitative RT-PCR (CFX96 Real-Time System, Bio-Rad) using primers targeting appropriate DNA sequences corresponding to ITGA6 promoter region. Primer sequences used in this work are listed in Supplementary Table 5. Statistical significances were calculated by two-tailed *t*-test.

### Quantitative reverse transcriptase–PCR

Total RNA was isolated using the RNA easy column kit (Qiagen) according to the manufacturer’s instructions. One microgram of total RNA per sample was annealed with anchored oligo (dT) for cDNA synthesis using M-MuLV Reverse Transcriptase (Thermo Scientific). qPCR was performed by using Brilliant III Ultra-FAST SYBR Green qPCR master mix (Stratagene, AH diagnostics Oy, Helsinki, Finland) and MX3005P (Stratagene), as described previously. Only primers resulting in a single peak in the melting curve analysis were used. The sequences of qPCR primers are listed in Supplementary Table 5. Results are representative of three independent experiments and difference. Significance was calculated by two-tailed *t*-test.

### *In vitro* luciferase assay

DNA fragment of ITGA6 promoter (chr.36:16,775,314-16,776,486) was inserted into the pGL3-Basic vector (E1751, Promega, Nacka, Sweden) upstream of Luciferase gene. A control plasmid pGL75 or the α6-promoter luciferase plasmid was co-transfected into MDCK cells and FOSL1-MDCK cells (100 μl of 1.5 × 10^5^ per well) using X-treme GENE HP DNA Transfection Reagent (06366236001, Roche Applied Science, Roche Diagnostics Oy, Espoo, Finland) in the 96-well white plate. After 48 h, Firefly and *Renilla* luciferase activities were measured with Dual-Glo Luciferase assay system (E2940, Promega). The experiments were performed in triplicate. Cloning primers are listed in Supplementary Table 3.

### Assessment of anoikis *In vitro*

WT-, K-Ras^V12^-, α6-KO/ K-Ras^V12^- or FOSL1-KO/ K-Ras^V12^-MDCK cells (1 × 10^6^) were seeded in polyHEMA pre-coated 10 cm plates at 37 °C for 24 h. The cells were scratched from plate and wash with cold PBS for flow cytometry analysis by using an Annexin V-fluorescein isothiocyanate/propidium iodide kit (BD Pharmingen, Becton Dickinson Oy, Vantaa, Finland; 556547). Caspase-3 activity was measured using western blotting of a cleaved-caspase-3 antibody and pro-apoptotic activity was detected using Bax antibodies (Supplementary Table 2).

### Cancer metastasis *in vivo* model

Animal protocols were approved by the University of Oulu Animal Care. The tail-vein of 7-week-old female 22 NOD.CB17-PrkdcSCID mice (six mice for each cell lines, except four mice for GFP-K-Ras^V12^ MDCK cells) were injected with 1 × 10^6^ cells (WT-GFP-, K-Ras^V12^-GFP-, α6-KO1/K-Ras^V12^-GFP- and FOSL1-KO1/K-Ras^V12^-GFP-MDCK cells) suspended in 150 μl of MEM (ThermoFisher, 11095-080). Upon killing on 15 days (except for three mice injected with K-Ras^V12^-GFP-MDCK cells, which had to be killed earlier due to their poor condition), lungs were dissected for *in vivo* optical imaging by IVIS Spectrum (PerkinElmer). To image lung metastases, part of the dissected lungs were embedded (Fisher Healthcare Tissue-Plus O.C.T Compound, 23-730-571, Fisher & Paykel Healthcare AB, Helsinki, Finland) for frozen tissue sections (Cryotome Leica CM3050S, Leica Microsystems, Immuno Diagnostic Oy LMS, Espoo, Finland). GFP-positive areas were determined by using Zeiss Axio Lab fluorescence microscope (Carl Zeiss Oy) and ImageJ software.^67^ In addition, the remaining parts of the dissected lungs were fixed in Bouin’s fixative for 24 h and embedded with paraffin for cross-sections and staining with hematoxylin and eosin solution. Tumor area was determined by using Leica microscope and ImageJ software.^67^

### Cell proliferation and migration

For cell proliferation assay, about 1000 cells per well were seeded into 96-well plates and tested with the XTT kit (11465015001, Roche) at designed time points by reading the absorbance at 450 nm, following the manufacturer’s instructions. Values were obtained from four replicate wells for each treatment and time point. For wound-healing assay, we used a culture-insert two-well in 35 mm μ-Dish (81176, IBIDI). First, cells were allowed to grow to confluency, after which the IBIDI-insert was removed using sterile tweezers. Time-lapse videos were recorded from 0 to 16 h. Results are representative of three independent experiments. Statistical significance was calculated by two-tailed *t*-test.

### Statistical analysis

Normally distributed samples were tested for significant differences with one-way analysis of variance between two groups. A Student's *t*-test (unpaired, two-tailed) was used to determine significant differences in normalized samples, unless otherwise noted. Error bars denote s.d. *P*-values<0.05 were considered as significant (*P<0.05, *P*<0.01 and ***P<0.001).
