# Supplementary material for: Oncogenic K-Ras upregulates ITGA6 expression via FOSL1 to induce anoikis resistance and synergizes with αV-Class integrins to promote EMT
Source: Oncogene. 2017 Jun 12;36(41):5681–94. doi: 10.1038/onc.2017.177 (PMC5658677; doi:10.1038/onc.2017.177)
Supplement: Supplementary Figures [file onc2017177x1.pdf]

*Zhang et.al.* Supplementary Figure 1.

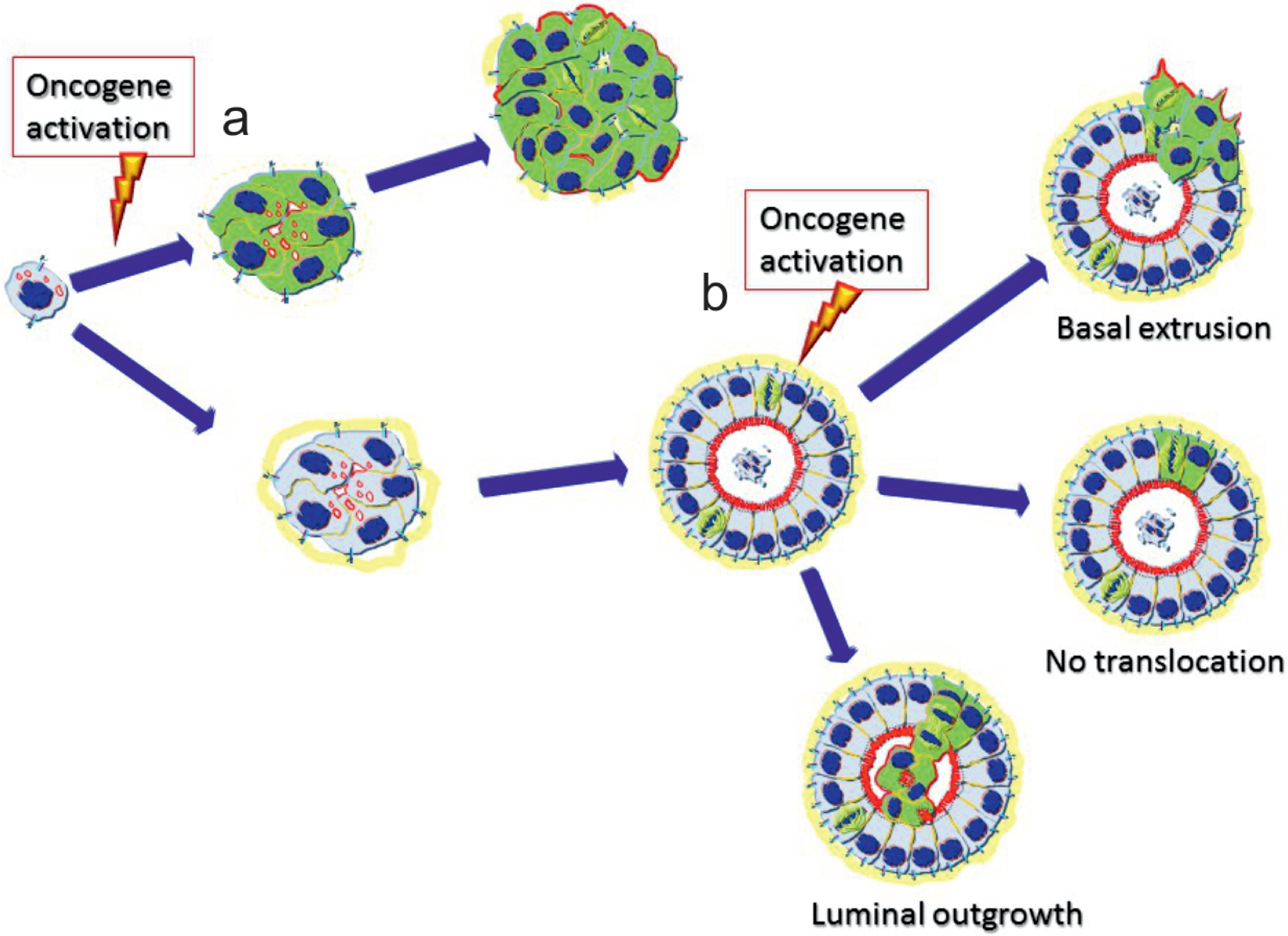

# *Zhang et.al.* Supplementary Figure 2.

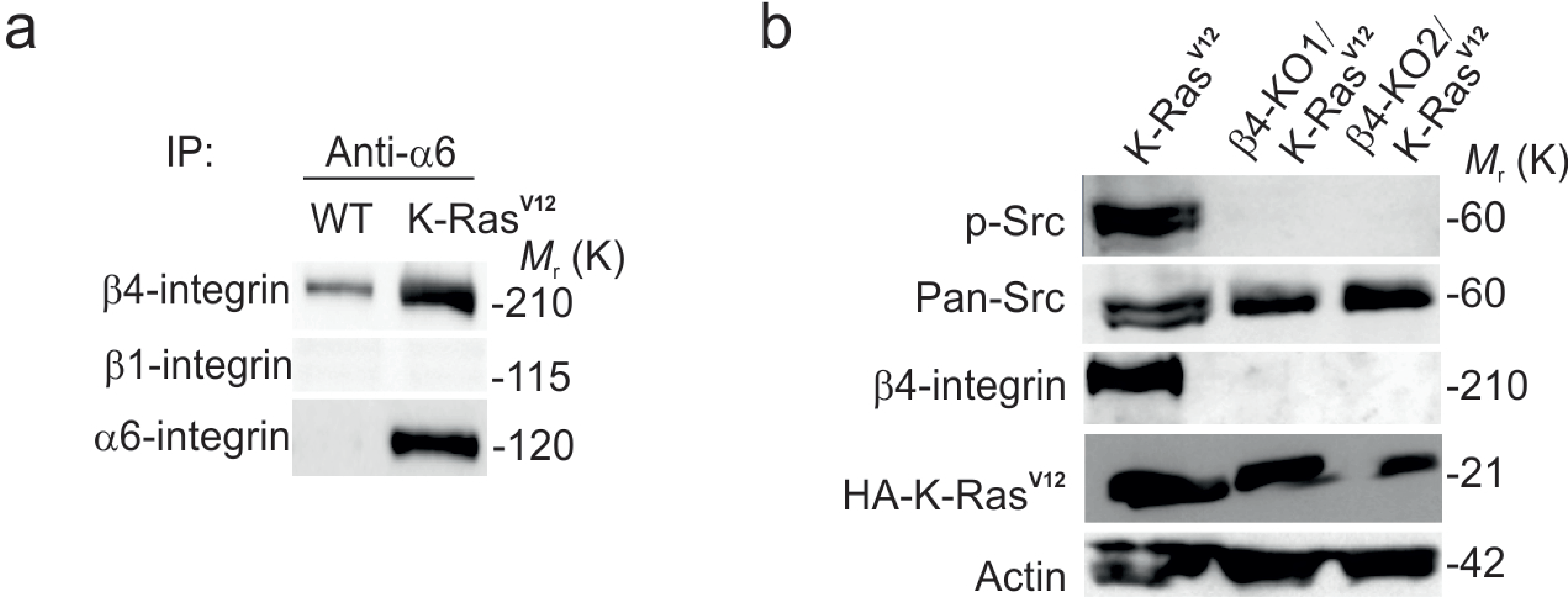

# *Zhang et.al.* Supplementary Figure 3.

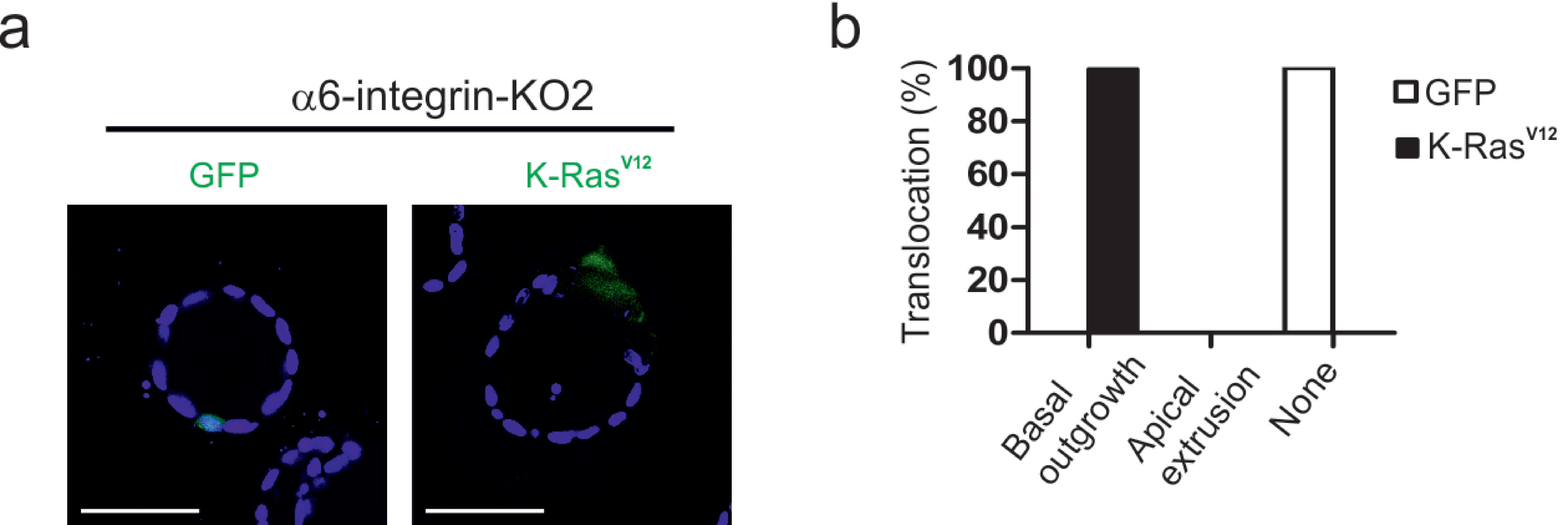

# *Zhang et.al.* Supplementary Figure 4.

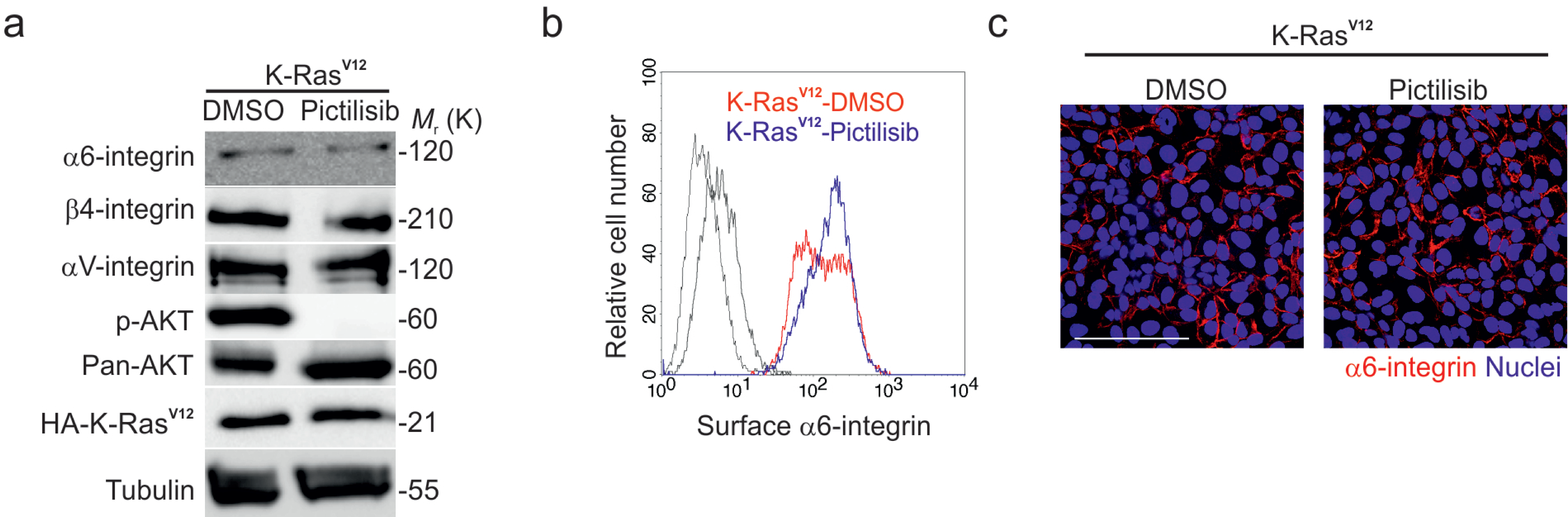

# *Zhang et.al.* Supplementary Figure 5.

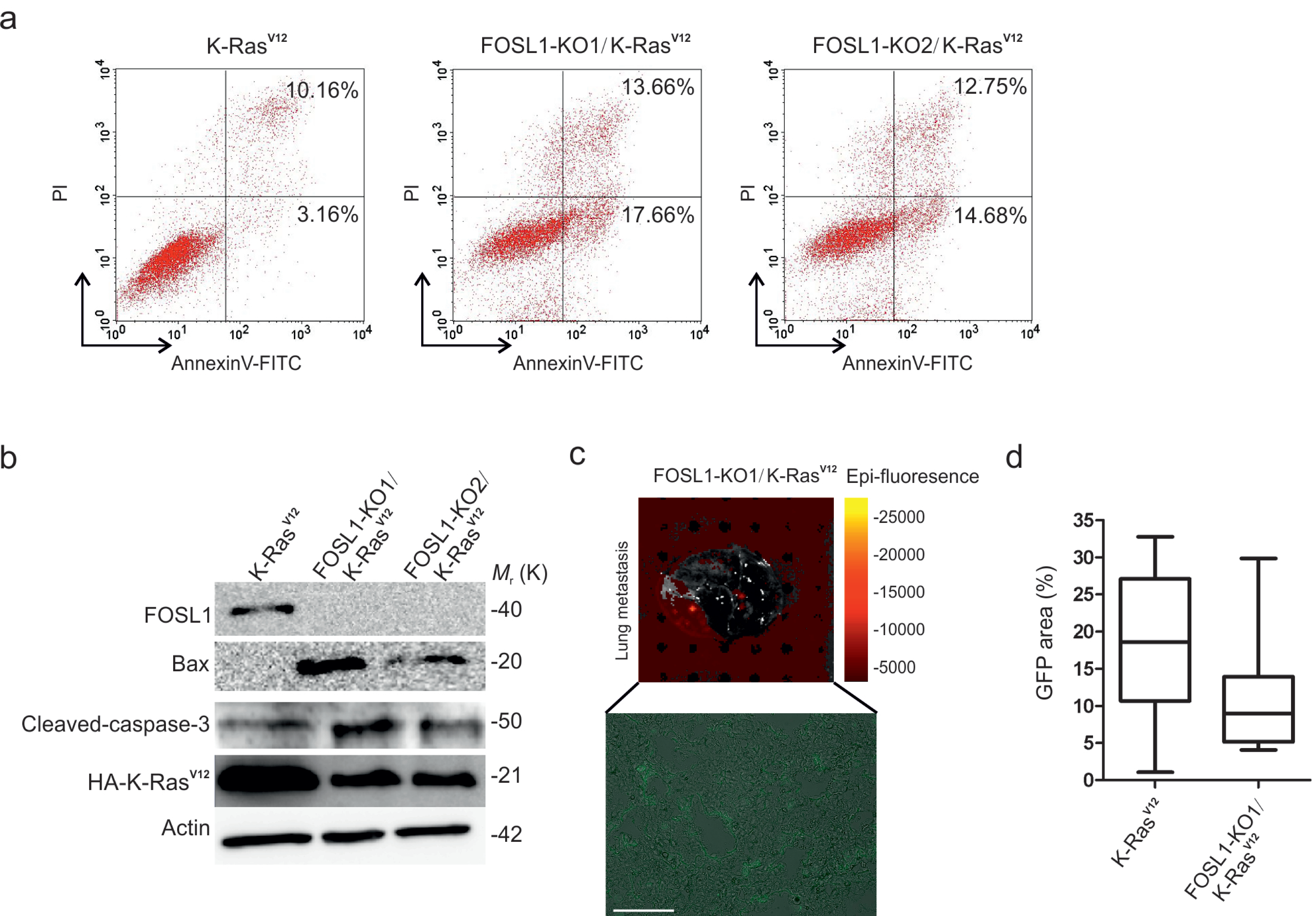

# *Zhang et.al.* Supplementary Figure 6

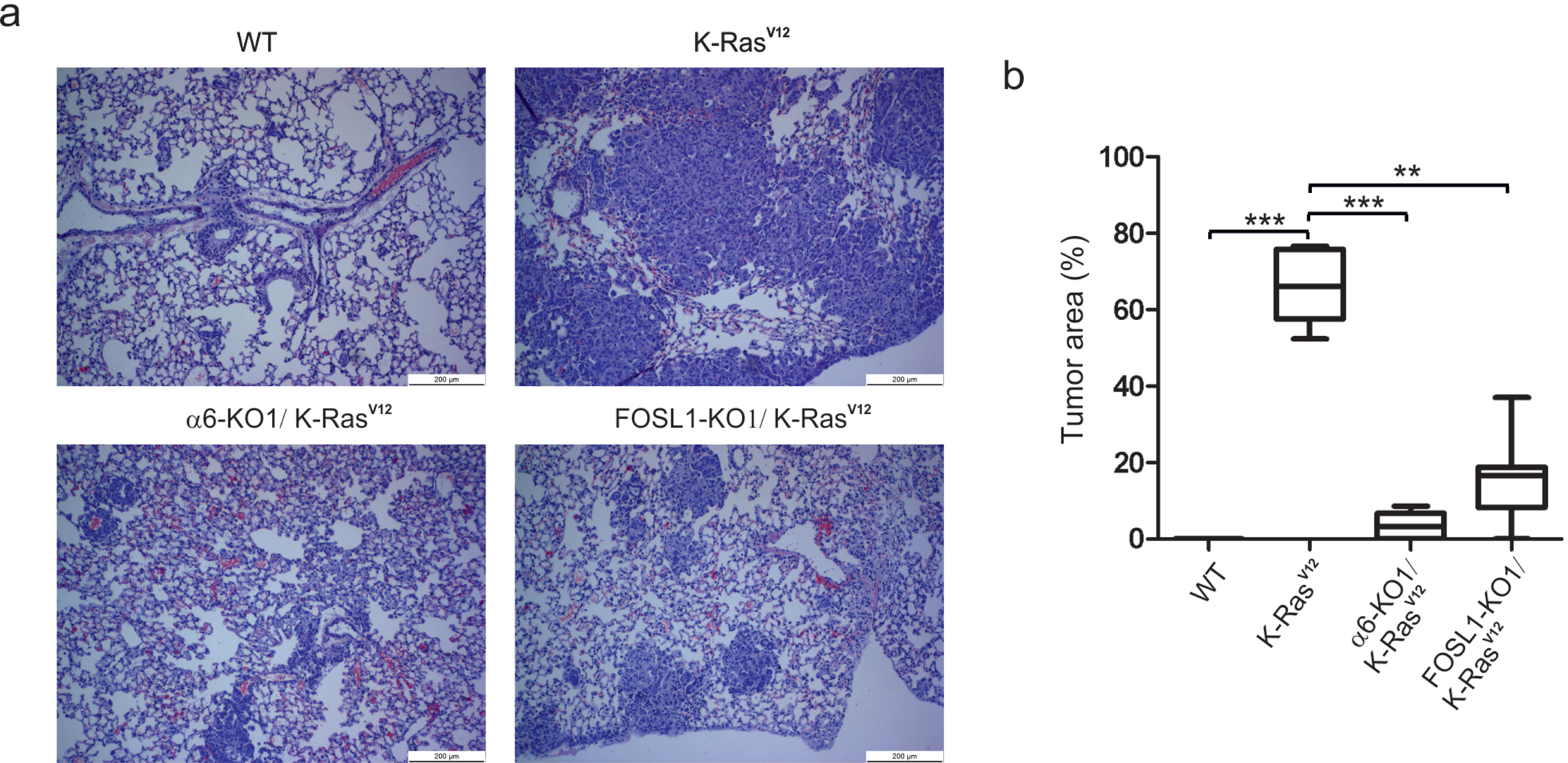

*Zhang et.al.* Supplementary Figure 7.

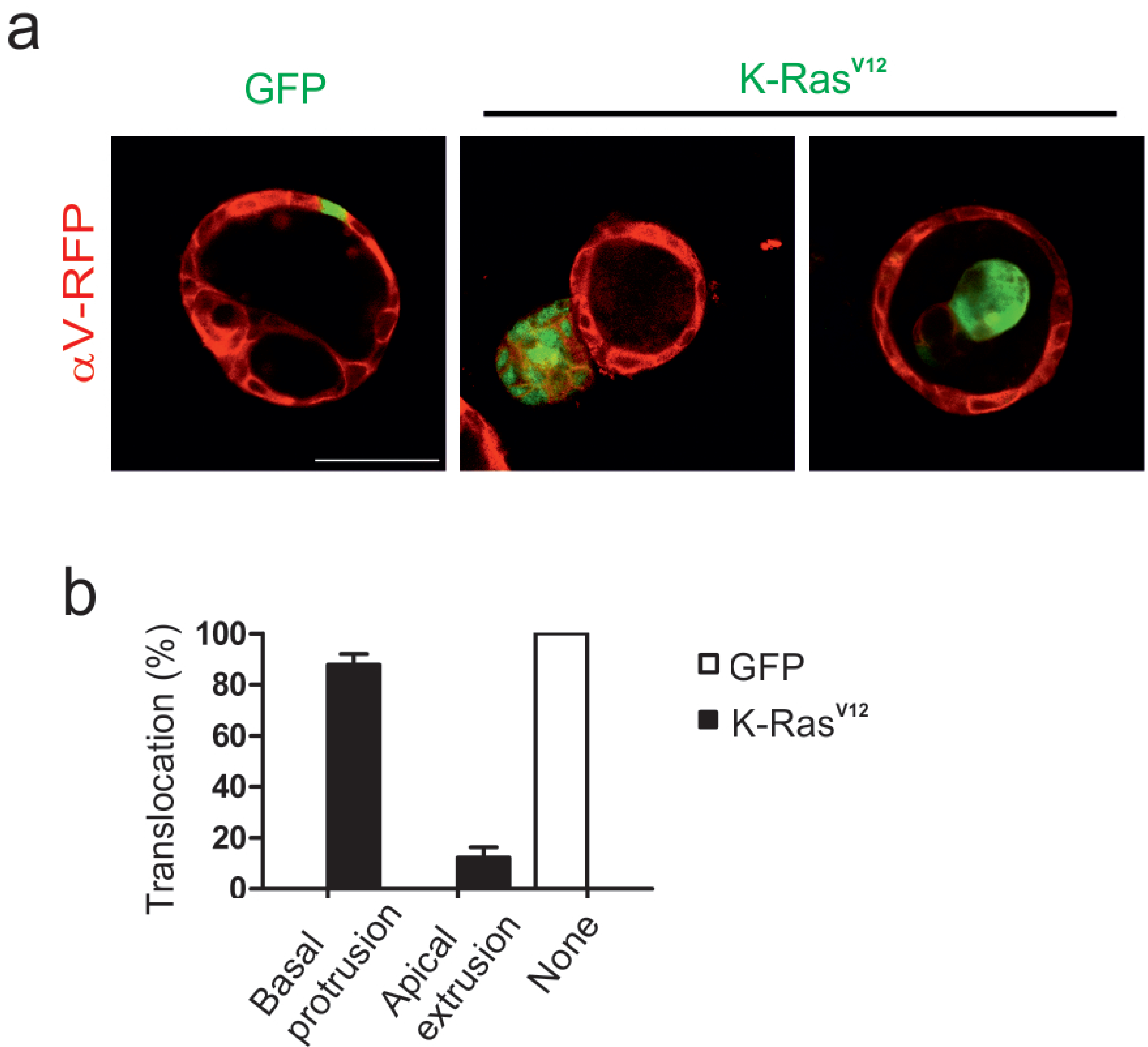

# *Zhang et.al.* Supplementary Figure 8.

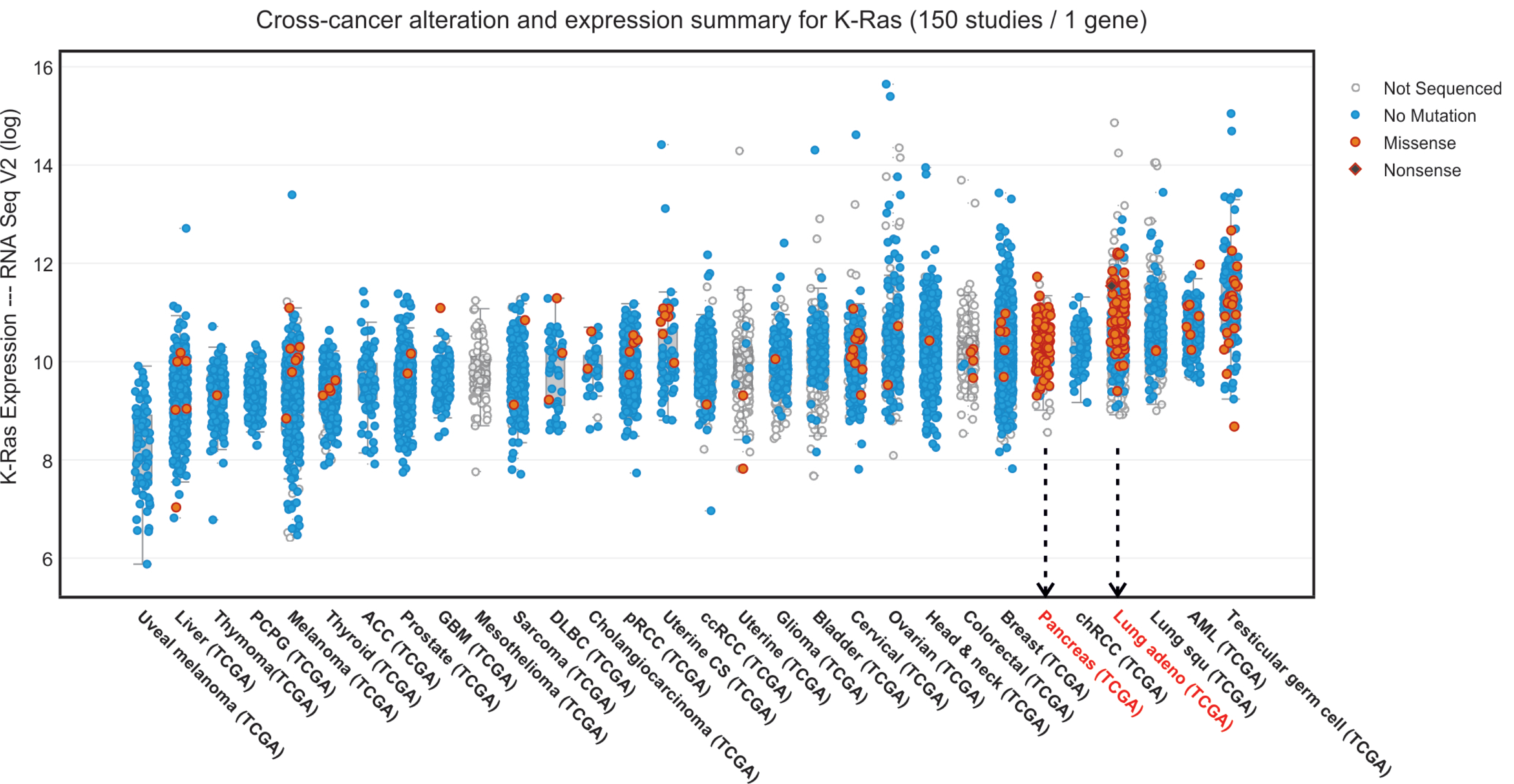

*Zhang et.al.* Supplementary Figure 9.

## Figure 3a.

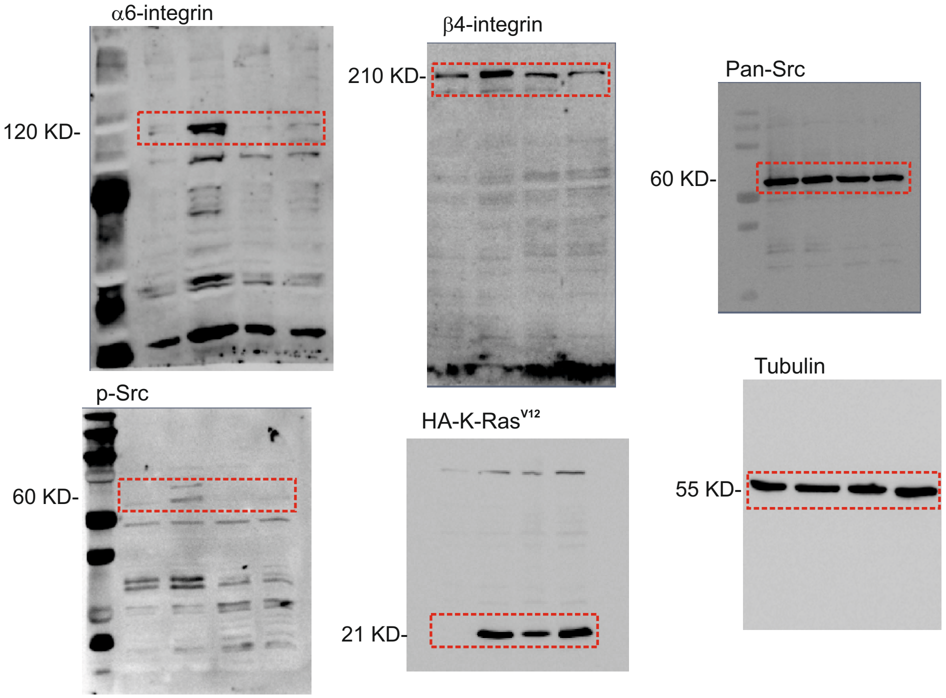


## Figure 4e.

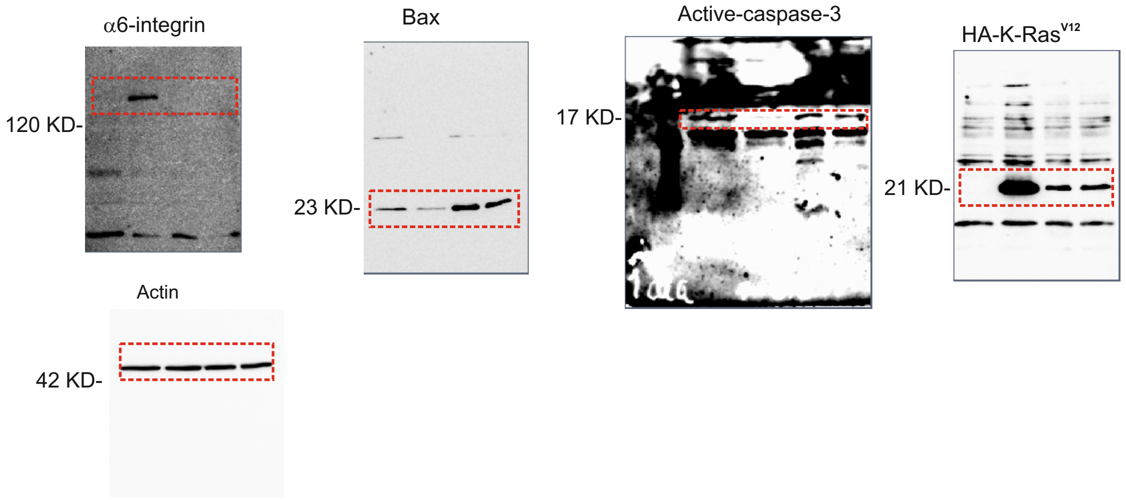


Uncropped original western blot full scans.

# Figure 5a.

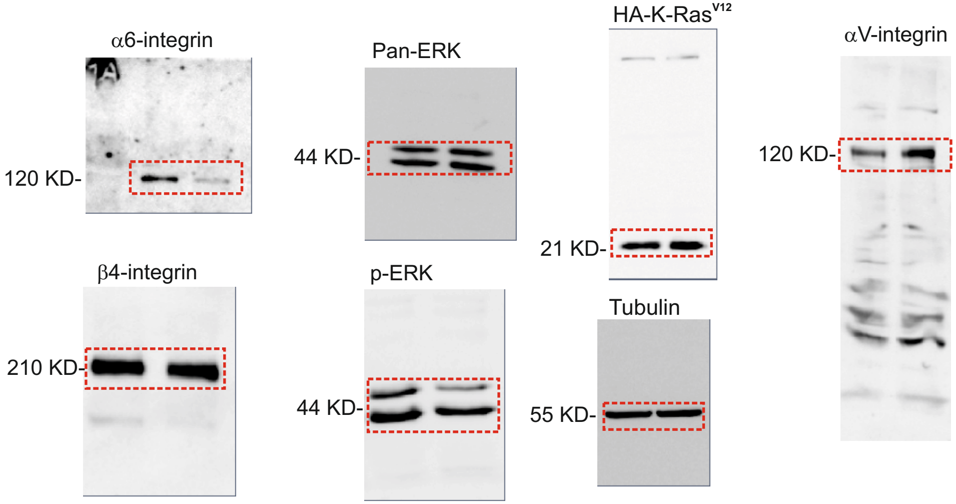


# Figure 5e.

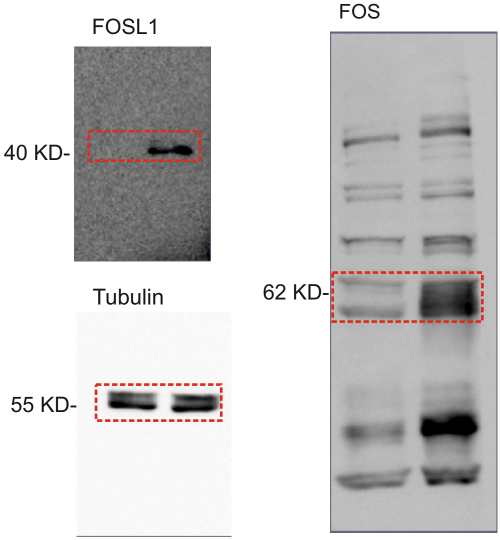


# Figure 5f.

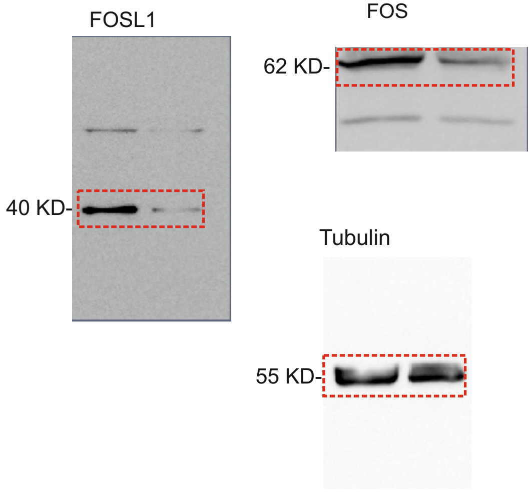


Uncropped original western blot full scans.

# Figure 5i.

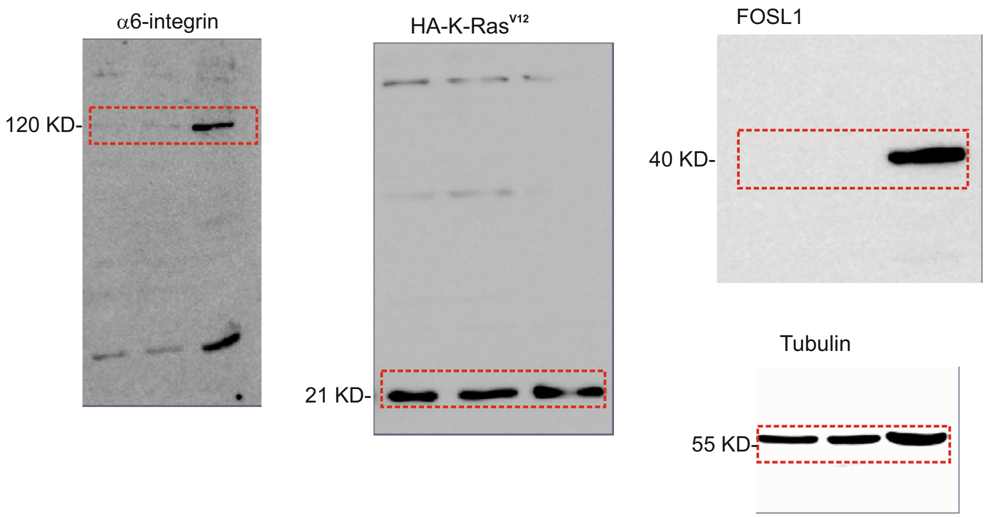


# Figure 6b.

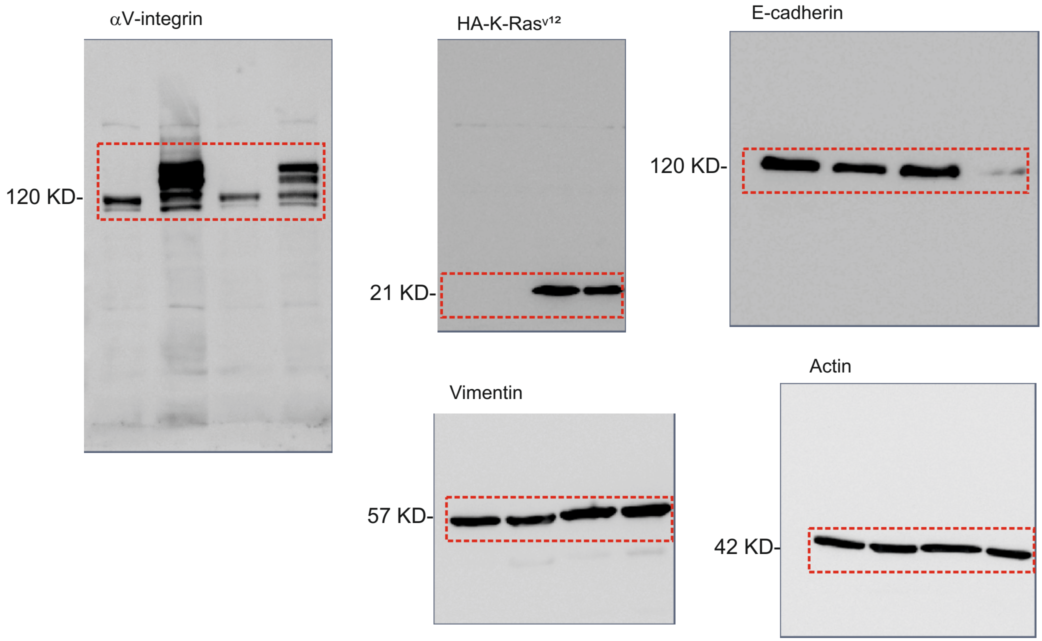


Uncropped original western blot full scans.

# Figure 7d.

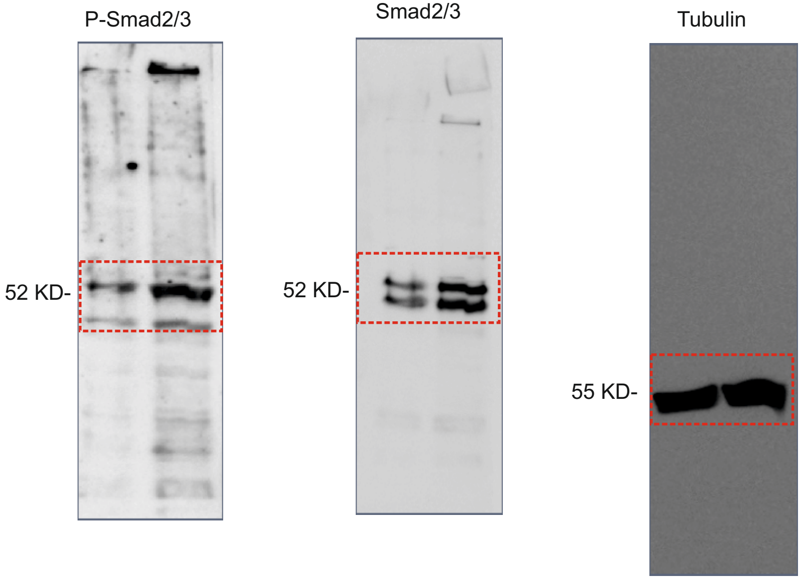


# Figure 7e.

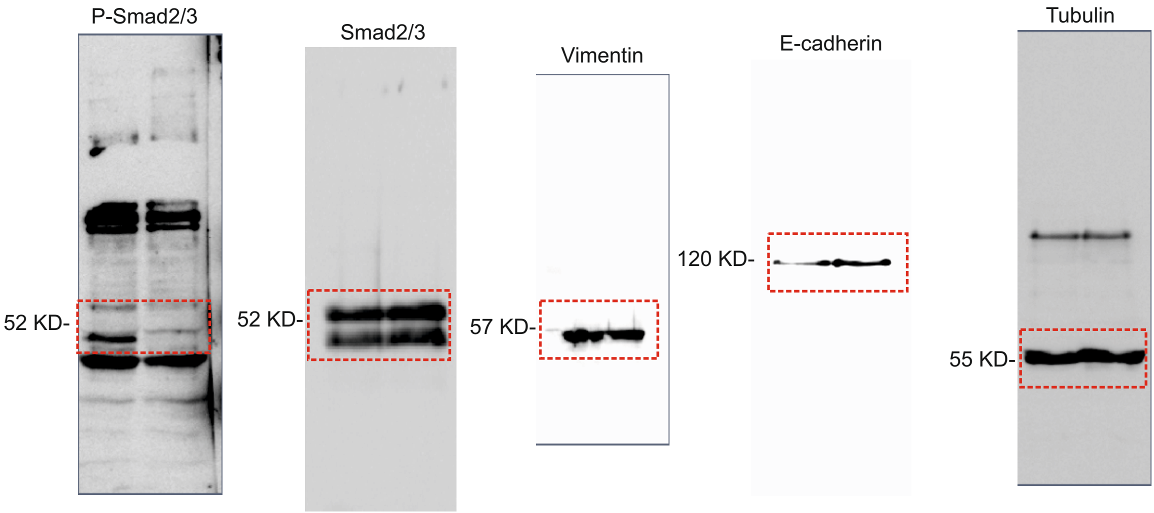


Uncropped original western blot full scans.

# Supplementary figure 2a.

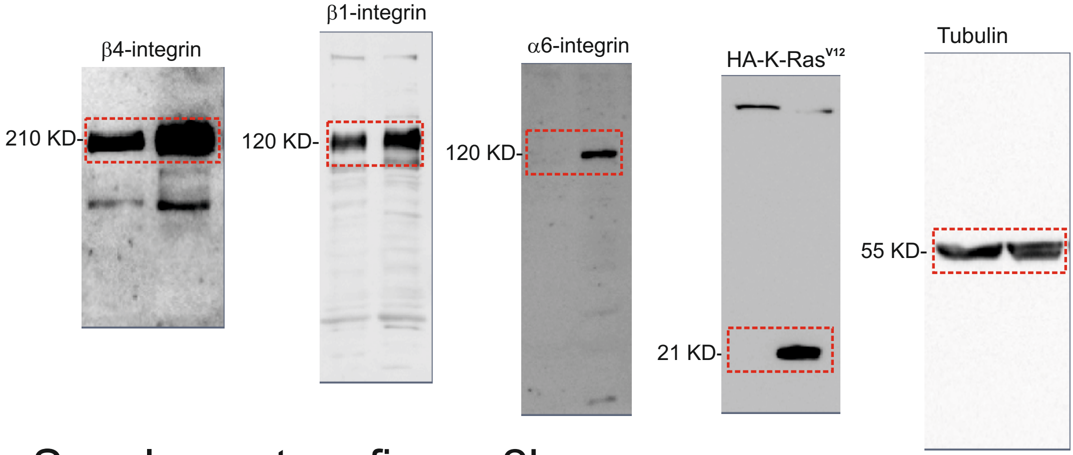


# Supplementary figure 2b.

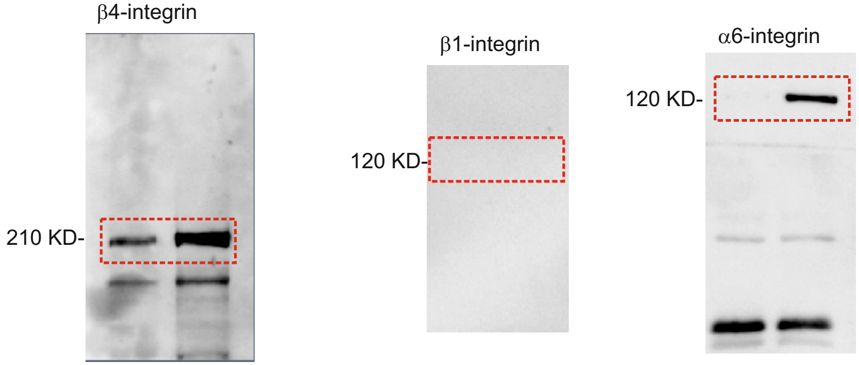


# Supplementary figure 2c.

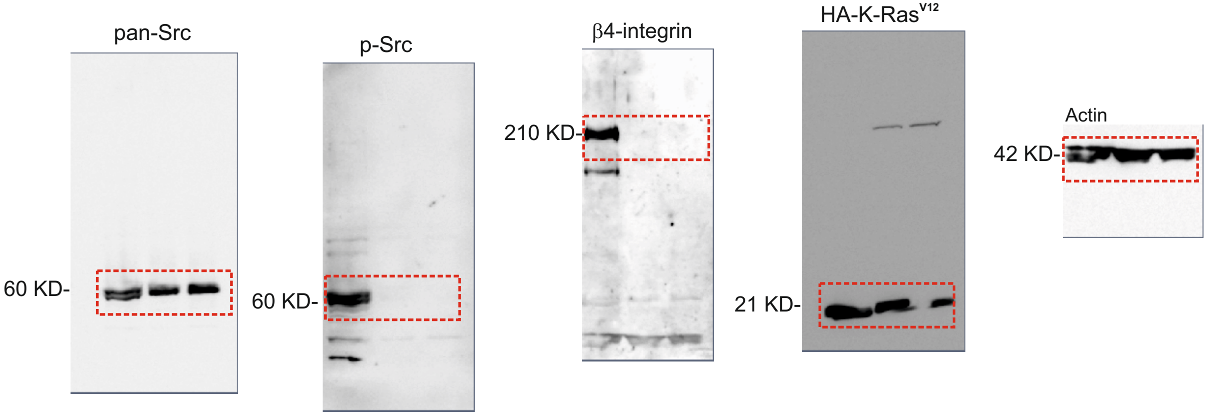


Uncropped original western blot full scans.

# Supplementary figure 3a.

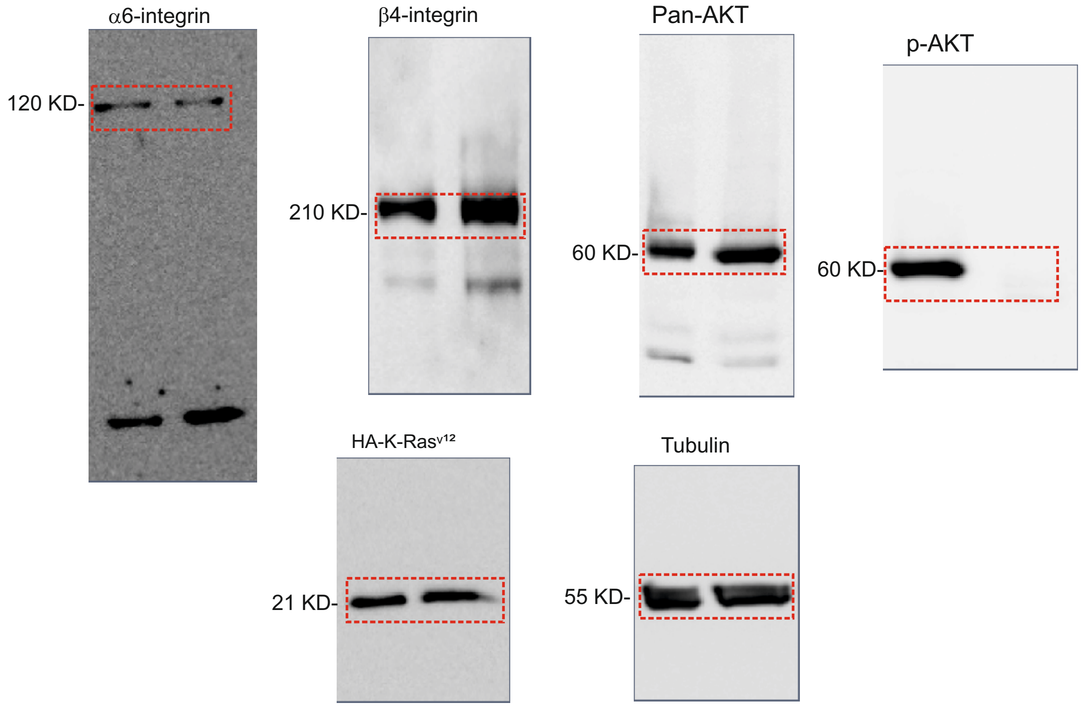


# Supplementary figure 5b.

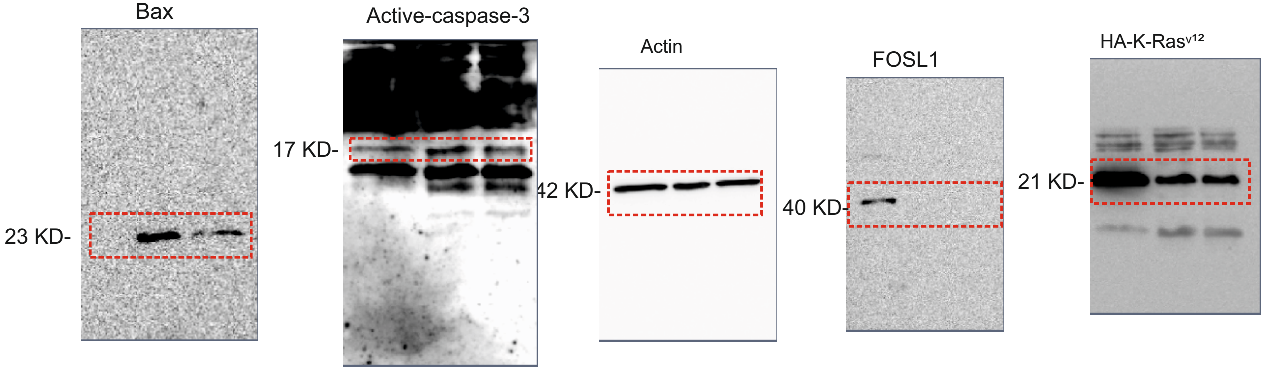


# Uncropped original western blot full scans.

## Supplementary Figure legends

**Supplementary Figure 1 A model of oncogene-mediated transformation in non-polarized versus polarized epithelial cysts.**

**(a)** Cell transformed by oncogenes prior to polarization can be modeled by seeding oncogene-expressing cells into 3D matrix where they show perturbed growth and form lumenless cell clusters or cysts with cells filling the lumen. **(b)** If cells are first allowed to grow in 3D matrix for 6 days to form polarized cysts they can then be transformed with limiting lentivirus concentrations to allow transformation of single cells within polarized cysts. Upon further culture for 4 days these transformed cells, depending on the oncogene, may continue to grow but remain polarized, or they may be extruded basally or apically (into the lumen). Luminally extruded cells may either survive or undergo anoikis.

**Supplementary Figure 2 α6β4-integrins is required for K-Ras$^{V12}$-induced activation of Src.**

**(a)** Immunoprecipitation of endogenous α6-integrin followed by immunoblotting with antibodies against β1-, β4- and α6-integrins. α6-integrins form preferentially a α6β4-heterodimer in both WT- and K-Ras$^{V12}$-MDCK cells. **(b)** Representative western blot analysis of K-Ras$^{V12}$ and two independent β4-KO/K-Ras$^{V12-}$MDCK cell line lysates using antibodies against β4-integrin, phosphorylated-Src (pTyr$^{416}$; p-Src), pan-Src, Hemagglutinin (HA)-tagged K-Ras$^{V12}$ (HA-K-Ras$^{V12}$) and actin.

**Supplementary Figure 3 α6-integrin expression is not essential for basal outgrowth or extrusion of K-Ras$^{V12}$-transformed cells.**

**(a)** α6-integrin-KO cells were grown in 3D cultures for 6 days to allow formation of polarized cysts followed a by single cell infection protocol as described in Fig. 1c. Representative images of clonal expansion of control (GFP only) and K-Ras$^{V12}$-transduced (K-Ras$^{V12}$; GFP-positive) α6-KO MDCK cells imaged 5 days after infection are shown. **(b)** Quantification of clonal expansion and polarized cell extrusion from three independent experiments (n=50 for each condition). Data are presented as the mean ± S.D. Scale bars, 50 µm.

**Supplementary Figure 4 K-Ras$^{V12}$ induced α6-integrin expression does not depend on PI3K pathway.**

**(a)** MDCK cells transduced with K-Ras$^{V12}$-expressing viral vector were cultured for 5 days in the presence of DMSO (0.01%) or Pictilisib (1 µM dilution in 0.01% DMSO) after which they were subjected to immunoblotting with antibodies against α6-integrin, β4-integrin, phosphorylated-AKT (pAKT, pSer473), total AKT (pan-AKT), HA (HA-K-Ras$^{V12}$) and tubulin. **(b)** FACS analysis of the surface expressed α6-integrin in mock (DMSO; red) or Pictilisib (1 µM; blue)-treated K-Ras$^{V12}$-MDCK cells. Histograms of unlabeled cells are shown in grey. **(c)** Confocal sections of mock (DMSO; red) or Pictilisib (1 µM; blue)-treated K-Ras$^{V12}$-MDCK cells stained for α6-integrin (red) and nuclei (blue). Scale bars, 100 µm.

**Supplementary Figure 5 Knockout of FOSL1 promotes anoikis in K-Ras$^{V12}$-MDCK cells. (a)** K-Ras$^{V12}$ cells and FOSLKO/ K-Ras$^{V12}$ cells were seeded onto polyHEMA-coated plates and allowed to grow for 24 h prior to FACS-based analysis using Annexin-V/PI staining kit. **(b)** Western blot analysis of apoptosis marker cleaved-caspase-3 and the pro-apoptotic protein Bax in polyHEMA-cultured (24 h) K-Ras$^{V12}$ cells and FOSL1-KO/ K-Ras$^{V12}$ cells. **(c)** Representative images of GFP-expressing FOSL1-

KO/ K-Ras$^{V12}$ MDCK cell metastases in lungs of immunocompromised mice. **(d)** Quantification of lung metastases as area of GFP-positive colonies relative to the total lung tissue area. Data is presented as the mean ± S.D. At least ten randomly selected slides were analyzed per each sample. Statistical significance was determined using two-tailed t-test. P-values < 0.05 are signified by (*), < 0.01 by (**) and < 0.001 by (***).

**Supplementary Figure 6 Metastatic capacity of K-Ras$^{V12}$-transformed MDCK cells is regulated by α6-integrin and FOSL1.**

**(a)** Representative images of hematoxylin and eosin stained paraffin sections of lungs from mice injected with GFP-expressing WT-, K-Ras$^{V12}$, and α6-KO1/K-Ras$^{V12}$-MDCK cells. Scale bars, 200 µm. **(b)** Quantification of lung metastases as relative area of the lung tissue [%] covered by tumor cell mass. Data is presented as the mean ± S.D. At least six randomly selected slides were analyzed per each sample. Statistical significance was determined using two-tailed t-test. P-values < 0.05 are signified by (*), < 0.01 by (**) and < 0.001 by (***).

**Supplementary Figure 7 Restoration of αV-RFP in K-Ras$^{V12}$ cells promote basal outgrowth in suppressive polarized epithelial microenvironment.**

**(a)** Representative images of αV-RFP-expressing MDCK cysts in which single cells were transduced either with GFP-only or GFP/K-Ras$^{V12}$-expressing viral vectors as described in Figure 1f. **(b)** Quantification of clonal expansion and polarized cell extrusion from three independent experiments (n=50 for each condition). Data are presented as the mean ± S.D. Scale bars, 50 µm.

**Supplementary Figure 8 Pancreatic Adenocarcinoma (PAAD) and Lung Adenocarcinoma (LUAD) have K-Ras high alteration and expression in 30 human cancers.** K-Ras gene expression and mutation data combined from 150 human cancers studies. Data was analyzed using cBioportal application[5,6] and shows both the relative expression levels of K-Ras (y-axis) and the mutation status of K-Ras in each individual sample (blue circle = wt K-Ras; orange circle = mutated K-Ras; white circle = no information).

**Supplementary Figure 9 Uncropped original western blot full scans.**

## References


1 Chen X, Long H, Gao P, Deng G, Pan Z, Liang J *et al.* Transcriptome assembly and analysis of Tibetan hulless barley (Hordeum vulgare L. var. nudum) developing grains, with emphasis on quality properties. *PLoS One* 2014; **9**: 1–13.

2 Trapnell C, Roberts A, Goff L, Pertea G, Kim D, Kelley DR *et al.* Differential gene and transcript expression analysis of RNA-seq experiments with TopHat and Cufflinks. *Nat Protoc* 2012; **7**: 562–78.

3 Myllymäki SM, Teräväinen TP, Manninen A. Two Distinct Integrin-Mediated Mechanisms Contribute to Apical Lumen Formation in Epithelial Cells. *PLoS One* 2011; **6**: e19453.

4 Huang Q, Whitington T, Gao P, Lindberg JF, Yang Y, Sun J *et al.* A prostate cancer susceptibility allele at 6q22 increases RFX6 expression by modulating HOXB13 chromatin binding. *Nat Genet* 2014; **46**: 126–35.

5 Cerami E, Gao J, Dogrusoz U, Gross BE, Sumer SO, Aksoy BA *et al.* The cBio Cancer

Genomics Portal: An Open Platform for Exploring Multidimensional Cancer Genomics Data. *Cancer Discov* 2012; **2**.

6 Gao J, Aksoy BA, Dogrusoz U, Dresdner G, Gross B, Sumer SO *et al.* Integrative Analysis of Complex Cancer Genomics and Clinical Profiles Using the cBioPortal. *Sci Signal* 2013; **6**: pl1-pl1.
