# Supplementary material for: Oncogenic K-Ras upregulates ITGA6 expression via FOSL1 to induce anoikis resistance and synergizes with αV-Class integrins to promote EMT
Source: Oncogene. 2017 Jun 12;36(41):5681–94. doi: 10.1038/onc.2017.177 (PMC5658677; doi:10.1038/onc.2017.177)
Supplement: Supplementary Tables [file onc2017177x2.pdf]

Supplementary Table 1 RNA-seq analysis (WT-, K-Ras^{12V}- and K-Ras12V/αV-RFP-MDCK)

P-value≤ 0.05, fold change ≥ 2

a. WT and K-Ras^{12V} -MDCK

up	down
TRIM36	SUOX
MAP3K5	ATP8B1
ENSCAFG00000000402	PDE7B
DPCR1	LRIG3
RGS17	SASH1
IL5	AKAP12
TRIB1	SYNE1
ST3GAL1	DLA88
OBSCN	DLA88
GRAP2	DLA-64
STEAP2	ZDHHC14
VEGFA	SLC44A4
FHL2	SOD2
PDK4	ENSCAFG00000000686
ETV1	TMEM74
OXER1	CELSR1
KLF4	DLA-DRA1
THAP5	DLA-DRB1
GJB3	DLA-DQA
AIM1	DLA-DQBC1
KLF15	CSF2
ENSCAFG00000003880	TAP2
KIAA1462	PSMB8
GP9	TAP1
ENSCAFG00000004348	DLA-DMA
PPM1N	IRF1
SERP2	DSE
PLK3	A4GALT
PLAUR	TAPBP
CEACAM28	ANXA13
42622	BAK1
SPRY2	SQLE
P4HA3	TGFBI
CBX4	PACSIN1
FCHSD2	LY6E
HBEGF	SCUBE3

b. K-Ras12V- and K-Ras12V/αV-RFP-MDCK

up	down
ZNF516	SERPINB8
GDF11	ERBB3
TSPAN8	IL23A
CELSR1	ATP8B1
FBLN1	TRIM36
TRPS1	C-MYB
SLC30A8	MAP7
CXCL14	TNFAIP3
TMEM200A	LRIG3
KLHL3	MGAT4B
DAPK1	AKAP12
NPTXR	CPM
MDGA1	C6ORF15
IFT27	NIPAL2
TRERF1	MIOX
ENSCAFG00000001866	GRAMD3
C7orf63	GRHL2
CKAP4	ADM2
KANK1	IRF1
GABBR2	FBXO32
PLEKHH2	SREBF2
COL15A1	SQLE
EEPD1	FAM84B
ENSCAFG00000003238	EGR1
FOXP2	FAM83F
RRM2	EXOC4
ODC1	MAPK13
ENSCAFG00000003565	PIM1
NRCAM	TTC39B
NRP1	ENSCAFG00000001616
ENSCAFG00000003792	MOB3B
ZEB1	ABCB4
EFR3B	MDR1
NDNF	TRPM3
PAK1	GTPBP2
TMEM163	VEGFA

NR1D2	GSDMD	IFT172	TEC
SH3BP5	DAPK1	LTBP4	GALT
DUSP6	PDGFB	THBD	TMEM151B
SOCS2	PLXNA4	MGAT5B	ENSCAFG00000002078
SPRY4	PODXL	SNX5	IL1RL1
NR3C1	MDGA1	ZEB2	NFIL3
PRKCDBP	CDC42EP1	FLRT3	ASNS
SNAI2	SMO	MKKS	ICA1
NR1H4	TSPAN33	SYT1	FAM83B
PLAG1	TTC39B	PLCL2	CNNM4
MALL	MLLT3	TMX4	OXER1
GRAMD1A	DMRTA1	PCNA	AGR2
GGH	PCSK5	COL4A1	ZNF667
IL20RB	APOL6	COL4A2	SLC2A1
GALNT6	ALDH1A1	SMAD9	EPS8L1
PROCR	DDX58	MORN2	EPCAM
HK2	KIF24	DDRGK1	RAPGEF5
PTK2B	KANK1	PKDCC	GPNMB
KLHL2	SAMD9L	HSPH1	OSBPL3
ANTXR2	TFPI2	NOP56	SKAP2
EVA1C	SLC1A1	STX2	TAX1BP1
HERPUD1	GPR110	ADAMTS9	CHN2
ADAM28	NCK2	TMEM74B	RASSF6
AGPAT9	PPP1R9A	ENSCAFG00000007045	GARS
TOX2	PKHD1	TM9SF4	RRAGD
DENND2C	SEMA4D	SNTA1	MACF1
ATP2B4	NPAS2	RCN1	AVL9
TOMM34	NINJ1	PALLD	THAP5
STK4	IGFBP7	AHCY	IFRD1
PAX2	TDRD7	REEP1	TES
TMEFF2	TSPAN13	CCNE1	COBL
CTSE	PLEKHH2	CADM2	STK17A
TFF1	MACC1	RBP4	INHBA
RGS2	ITGB8	RHOBTB3	EPHA1
DENND5B	EPAS1	PROCR	IRF3
CCND1	MSH2	MAP1B	GAB1
TNNT2	COL12A1	CYP2C21	STOM
SLC37A2	GPNMB	PLOD2	ENSCAFG00000003874
ROBO3	OSBPL3	EPB41L1	ENSCAFG00000003880
MMP-14	MYH14	ZNF704	PLEKHA4

PTPRC	ENSCAFG00000003238
K-RAS	ZNF618
SNAI1	RSAD2
FCGR1A	TNFSF15
IRF2BP2	TSPAN12
FAM65C	STK17A
MNDA	FCGRT
UBASH3B	TBC1D9
CD86	OSR1
ERN1	WDR35
LAMB3	ENSCAFG00000003792
DHRS9	ENSCAFG00000003845
CA5B	RHOB
SLC26A6	KLHL29
C2CD2L	PRKAR2B
IL10RA	SLC5A9
ITGA6	PARP12
EGR2	PDZK1IP1
GLUL	HS6ST1
FOSL1	NOX4
NRP2	NUP210
LAMC2	NINL
SIK3	PRSS23
HIP1	TSC22D1
ETV5	PRR2
COX-2	IGSF23
PLA2G4A	HID1
SRGN	TENM4
SYVN1	NCKAP5
CEACAM1	HRH1
PEA3	XYLB
ABLIM2	INSIG1
TNFSF18	MGAT5B
SLC11A1	CAPN14
DNM3	DGAT2
TNIK	GDPD5
PLD1	GPD1L
SDF2L1	LGALS3BP
BAIAP2L1	FLRT3
C10orf11	FARP1
FAM220A	KIF5C

EDIL3	RAB19
VCAN	PDZK1IP1
ADAMTS1	ABTB1
NDRG3	ZC3HAV1
CTSO	KIAA1217
PDGFC	PPM1N
DSN1	PPP1R13L
SOGA1	SYTL2
SAMHD1	GPRC5C
PLEKHA7	TMCC1
FRAS1	RELB
ARL2BP	SERP2
PPP1R16B	NOS3
FAM83D	TRANK1
ADAM28	LLGL2
PLCG1	KCTD14
VDR	KRTCAP3
SRSF6	VILL
IFT52	LMO7
MYBL2	INSIG1
ENSCAFG00000009397	SFMBT2
SLC39A14	SCEL
E2F8	FOSL2
CBR3	CIDEC
LRRK2	C1orf210
CAP2	RNASE4
WRB	RNASE6
TMEFF2	RNASE1
TFF1	MBNL2
CDON	ZFP36
LGR6	LMCD1
FSTL1	HBEGF
SDC3	MYADML2
PTGIS	SPINT2
SCN9A	MGAT4C
AIP	EIF2AK2
MFGE8	PKP3
NGEF	ENSCAFG0000000667 9
PITX2	NR1H4
SOX2	ZFAND4
MNDA	PLK2

B4GALNT3	BHLHE40
ZBTB18	RNF213
NEDD4	CXXC5
EPHA2	IDO2
TNS4	IDO1
SERPINE2	SATB1
PPP1R1B	TACC1
GALNT16	FGFR1
HEPH	MGAT4C
DNAH2	EIF2AK2
ERGIC1	QPCT
KDM6B	CDC42EP3
TLN2	SMAD9
FOS	DCLK1
SHC1	SEMA3E
TLE3	ARHGAP26
SYT4	P2X2
DEGS2	NRG1
NCAD	NTN4
MRP1	ARID5A
KLHL13	ENSCAFG00000006550
DOCK11	ENSCAFG00000006722
ITGA2	ADAMTS9
DLGAP1	MAP3K1
DAB2	ST5
GPC3	ENSCAFG00000007045
NPR3	PPP2R3A
MAP7D3	ENSCAFG00000007099
MYO10	ENSCAFG00000007115
BGN	ASAH1
MKNK2	ANKRD1
SSTR5	TCP11L1
ERRFI1	DEPDC7
PIK3CD	TLR3
S1PR1	RCN1
CCBL1	SORBS2
LMO4	PALLD
NQO1	ADM
CTH	MPPED2
7SK	SNX25

ATRNL1	MTUS1
DALRD3	LSR
DHRS9	HPN
GRK5	TMEM87B
MAP3K6	SLC16A12
PHYHIPL	LATS2
FAM13C	ANKRD1
HTRA1	PSD4
GPC1	ENSCAFG00000007307
QSOX1	EPB41L4A
ARHGDIB	EIF2AK3
CDC25A	KRCC1
ENSCAFG00000013064	NUDT12
ENSCAFG00000013158	VAMP5
FZD9	CELA1
MRC2	OCLN
IVNS1ABP	YPEL4
LEPREL1	ENSCAFG00000008181
CLDN16	KIF13B
IQCJ-SCHIP1	LIMA1
FN1	SORBS1
HTRA3	CP
GOLIM4	VEGFC
NID2	MMP15
FGFR3	KIFC3
MMP1	SHROOM3
TIMP1	MADD
SMTNL2	SOWAHB
NPTX2	TMEM144
CREG1	CCNG2
LIPA	ENSCAFG00000008716
IQSEC3	PLLP
EXO1	ANXA3
PADI2	ADCY6
ONECUT1	RASGEF1B
LEPREL4	OAS3
EPHA4	RIPK2
IGFBP4	HERPUD1
SNCG	RASAL1
SERPINE2	DYSF

DUSP2	CELA1
PRSS22	STOX2
CD55	ERAP1
ABCA8	UBE2L6
ENSCAFG00000028542	DCLK2
PPP1R3C	SLC43A3
RND3	ELL2
WNT5A	MSC
ENSCAFG00000029384	TRPA1
GADD45A	FDFT1
ENSCAFG00000029566	PLOD2
ACER2	ENSCAFG00000008154
IFNE	HNF4G
DUSP1	CXADR
ENSCAFG00000029914	ENSCAFG00000008335
DNAJB9	ENSCAFG00000008338
LRAT	SCARA3
ENSCAFG00000030495	ZNF704
DUSP5	EDIL3
ZBED2	MYL9
ENSCAFG00000030746	MMP15
ENSCAFG00000030791	CTSO
EREG	SCARB2
ENSCAFG00000031502	PLEKHA7
HPCAL1	THBS1
ENSCAFG00000032259	PLCH1
ENSCAFG00000032276	FRAS1
	CCL17
	CX3CL1
	TGM2
	PIK3AP1
	VEPH1
	KIAA1755
	LBP
	DDX60
	DYSF
	ARSB
	LOXL2
	MRP2
	RCAN1
	SPP1

TTYH3	C15orf52
ENSCAFG00000016475	HPSE
FGFR4	CDS1
GPR125	UBTD1
POLR2A	ANKRD2
GALNT16	ST3GAL6
C17orf96	CREB3L1
CPLX2	ESRP1
FUS	SPINT1
GNGT2	SORBS3
SPDL1	CHAC1
SLIT3	PTPN13
CYFIP2	SLC10A6
GPR68	MFSD6
NEO1	DENND2C
SEMA7A	ATP2B4
ALDH3A1	DSP
ENSCAFG00000018321	PLEKHA6
CD320	WFDC2
CACHD1	PRICKLE1
RFX2	ITGB6
JUN	WSB2
C1orf233	VSIG10
THOC6	PEBP1
AGRN	KCHIP2
FOXF1	ST14
FNBP1	ZNF697
TGFBR3	IGSF5
LRRC8C	TEX264
FHOD1	NFKB2
EXOC3L1	RNF144B
SNORA73	MBOAT1
7SK	RIPK4
ABCA2	BARX2
GAS1	CNNM2
CTXN1	PHLDB2
ITPR1	IFIH1
ENSCAFG00000023798	SLC37A1
FASTKD5	BSDC1
CA2	FGD4
IGFBP6	ELF3

BMP1	FAM171B	SLC16A14
WFDC2	ENSCAFG00000028727	MANBA
HERC6	RAB6B	SLC12A7
EDN1	ENSCAFG00000029117	TC2N
IGSF3	WNT5A	ADRA2A
CD101	TGIF2-C20orf24	LSAMP
KSR2	ENSCAFG00000029966	ARHGAP31
ITGB6	ENSCAFG00000030443	NPNT
RFC5	RPS19BP1	TINAGL1
GMPR	PID1	WIPI1
CD40	GLIPR2	SERINC2
DUSP8	CADM4	ROBO3
NUAK2	OAF	FABP3
WRB	BMP2	CASP7
CD81	LGALSL	ALDH3B1
SNRPN	GNG2	NR5A2
CCDC64	CNRIP1	GALNT3
MX2	SNAPC2	DAO
MX1	HPCAL1	ADAP1
RNF144B	FAM46A	ITPR2
CNNM2	FXYD6	FICD
DYNLL1	THEM6	K-RAS
DHCR7	SRM	ABLIM1
IFIH1		C1orf116
FAM65B		ISG20
CCL20		AP1G2
CCDC80		PARD6B
GRB14		SC4MOL
DAPP1		BCAS1
MARCKSL1		IGSF9
ELF3		SESN2
ENSCAFG00000010717		KLHL24
C2orf72		PCK2
SLC12A7		KIAA1598
TGFBETA2		LAMB3
TMTC1		PARP9
TC2N		DTX3L
FBLN5		CFLAR
LSAMP		SPTBN2
IGSF11		ATF3
ARRDC4		NFATC4

NPNT
SPATS2L
UBA7
FSTL1
DAO
SLCO3A1
HDHD1
DISP1
TXNIP
PTGIS
EGF
MFGE8
MID1
ADRBK2
SC4MOL
NFATC2
TIFA
SORL1
BCAS1
ASB9
KIAA1598
PARP9
DTX3L
PARP15
PARP14
KIF7
WDR6
CTSK
TRAF5
UGT8
KALRN
PRSS12
BICC1
ATF3
FGFR2IIIC
ITGB5
MGST1
GPC1
CYBRD1
SELENBP1

AIM1L
CLDN4
CLDN3
F11R
SELENBP1
PLXNB1
CGN
SNX27
TMPRSS4
PLA2G3
GSG1
GPRC5A
LAMC2
IGF2BP2
HSPB1
COX-2
SQRDL
ENSCAFG00000013770
PLA2G4A
ADORA2A
APOBEC1
LPP
TM7SF2
C11orf85
ENSCAFG00000014043
MID1IP1
CTSH
OSBPL1A
CDC42BPG
PDE1A
ENSCAFG00000014321
PTPN6
ENO2
ERO1L
LPAR5
DNM3
BIRC3
PLAU
PLEKHG6
FGFBP1

ANK3
SEC14L2
MUC20
FXYD2
TNK2
RHOBTB1
GRHL3
RNASEL
ID3
LUZP1
RGL1
NFKBIA
MOV10
PDK3
CLEC7A
POLA1
B2M
ENSCAFG00000013668
MYL4
RTP4
PAI-1
ARNT2
TM7SF2
SYTL5
LEPREL1
CLDN16
CRYAB
MB21D2
FZD2
C1R
IKZF2
PDE1A
ITGAD
EPHB2
B3GALNT1
EXPH5
C1S
PTPN6
BCHE
TNS1
ITGAV

SDF2L1
BST1
SLC3A2
DAAM1
PAPSS2
TMEM30B
ARHGEF10L
EVC2
SFTPD
ZBTB18
SYNE2
FAM214A
CXCL16
JUP
ENSCAFG00000015931
FADS2
PLEKHG3
SLC2A4
AP1S3
ENSCAFG00000016252
CGNL1
MYZAP
ARAP2
TMEM51
PPP1R1B
MAP3K9
OPHN1
STARD8
ZFYVE1
ERGIC1
KDM6B
NPC2
LRP
MUC1
FOS
JDP2
WWC1
YPEL3
NUPR1
IGDCC4
TMEM63C

IFI35
HSPG2
CASP12
ECE1
RPL22L1
ATP8B4
FGFR3
MMP13
RARRES3
BIRC3
TAPBPL
FGFBP1
TNFSF10
DHX58
SFTPD
RHOJ
SYNE2
FAM213A
CXCL16
NSUN7
WNT5B
FSCN1
SHROOM4
SNCG
ENSCAFG00000016170
ENSCAFG00000016252
TTYH3
KIAA0247
CASZ1
PRR15L
CA12
NFIX
TRIM46
ACSF2
EPN3
MYH10
STON2
FLRT2
GPR68
ENSCAFG00000017632
SCNN1B

HOOK2
MYH10
ADRA1B
SEL1L
TOM1L1
USP43
LDLR
AP1M2
FAM211A
GPRC5B
DSG3
SPECC1
TMC7
KLHL13
CAMSAP3
ESM1
FST
ITGA2
RAB11FIP4
DLGAP1
PDE4B
SH2D3A
LGALS9
INADL
ENSCAFG00000018826
MBNL3
NPR3
MYO5B
DHCR24
LIPG
NXN
STAP2
PPL
VASN
CCDC64B
ARHGEF16
ENSCAFG00000019801
NTNG2
IRF8
CMIP
CDYL2

CLMN
SLC44A2
NEO1
FHOD3
CD276
EVL
SEMA7A
C19orf66
B4GALT6
TMC5
CD74
IL13RA2
ARHGEF37
ENSCAFG00000018385
ENSCAFG00000018572
OXCT1
ARHGAP28
C3
TUBB4A
LGALS9
OSMR
LIFR
MKL2
GPC4
PIPOX
CDH6
DHCR24
GABRA3
AGRN
ISG15
GADD45B
FAM213B
CAMTA1
DENND2D
COL5A1
OLFM3
GBP6
PSMB10
IFI44
SNORA73

CCBL1
FA2H
TF
HSPA5
CYR61
MCOLN3
CDH3
CDH1
ESRP2
DUSP2
SOWAHC
KRT13
OAS1
PRSS22
KRT19
SULT1C4
ENSCAFG00000023754
SGPP2
PTGFRN
IGFL3
CLDN9
PRRG2
SERPINB5
ABCA8
ENSCAFG00000025389
DHRS2
IGJ
TMPRSS11E
CMPK2
ABHD17C
PPP1R3C
CSRP1
RND3
KCNK5
ENSCAFG00000029384
HAS2
CLIC2
GADD45A
ATF5
FOXN2

OAS2
ERBB4
CYP4A37
HAVCR1
OAS1
VNN1
ENSCAFG00000023625
LOXL1
ENSCAFG00000024641
KIF21B
FOLH1
CA2
ENSCAFG00000025394
SNORA31
TDRP
TMPRSS11E
FAM171B
PPAP2B
CMPK2
NREP
TRIB2
TIMP3
CLDN1
BASP1
CLIC2
B3GALT5
BMF
POU3F2
STXBP6
MMD
PAQR8
BAMBI
ENSCAFG00000030283
LTB
SERPINB9
PID1
IL22RA1
PSMB9

ACER2
MSRB3
DUSP1
TSC22D3
DNAJB9
DDAH1
ENSCAFG00000030095
ENSCAFG00000030204
C3orf52
ENSCAFG00000030354
DUSP5
PPP1R15A
ARHGAP8
FDPS
SLC25A29
ENSCAFG00000030791
IDI1
LIF
EMP1
YPEL5
KRT14
TMEM86A
SPINK6
cfa-mir-147
ENSCAFG00000031502
ENSCAFG00000031509
CRADD
STC2
C6orf132
NIPAL1
C11orf52
ENSCAFG00000032099
ENSCAFG00000032171
OLFM1
ENSCAFG00000032276
DDIT4
WNT7A

CA13
PXDC1
RALA
ENSCAFG00000030956
SERTAD4
SH2B2
ENSCAFG00000031299
BST2
SPINK6
PMEPA1
GNA14
WNT9A
ICAM1
GLIS3
LGALSL
IFIT3
GNG2
ENSCAFG00000032102
ENSCAFG00000032171
DLA-12
CLEC2L
PDGFD
THEM6

Supplementary Table 2 Antibodies

No.	Name	Target	Company	Cat. No.	Experiment
1	Anti-Human CD49f Clone GoH3	Human α6-integrin	BD Pharmingen	555734	FACS, IF
3	Anti-Integrin alpha 6 antibody	Human α6-integrin	Abcam	ab97760	WB
4	Anti-Integrin beta1 antibody	Human β1-integrin	Millipore	AB1952	WB
5	Anti-Integrin beta 4	Human β4-integrin	Abcam	ab-29042	WB
6	Anti-Integrin alphaV Antibody	Human αV-integrin	Millipore	AB1930	WB,IF
7	Anti-HA	HA-tag	Sigma-Aldrich	H9658	WB,IF
8	Monoclonal Anti-β-Tubulin	Human β-Tubulin	Sigma-Aldrich	T4026	WB
9	Phospho-Src Family (Tyr416) Antibody	Tyr419 of human Src	Cell Signaling Technology	2101	WB, IF
10	Src (36D10) Rabbit mAb	Human Src	Cell Signaling Technology	36D10	WB, IF
11	Cleaved Caspase-3 (Asp175)	(Asp175) in human caspase-3	Cell Signaling Technology	9661	IF
12	Phospho-p44/42 MAPK (Erk1/2)	Thr202/Tyr204 of human p44 MAP kinase	Cell Signaling Technology	9101	WB
13	p44/42 MAPK (Erk1/2)	C-terminus of rat p44 MAP Kinase	Cell Signaling Technology	4695	WB
14	FOSL1 antibody	Human FOSL1	Developmental Studies Hybridoma Bank	PCRP-FOSL1-1E3	WB, ChIP
15	c-Fos antibody (H-125)	Human c-Fos	Santa Cruz Biotechnology	sc-7202 X	WB, ChIP
16	Vimentin	Human Vimentin	Dako	M072529-2	WB, IF
17	Mouse Anti-E-Cadherin	Human E-Cadherin C-terminal Recombinant Protein	BD Transduction Laboratories	610182	WB, IF
18	Phospho-Akt (Ser473) Antibody	Ser473 of mouse Akt	Cell Signaling Technology	9271	WB
19	Akt (pan) (C67E7)	Mouse Akt	Cell Signaling Technology	4691	WB
20	Anti-Caspase-3	Cleavage site of human caspase 3	Sigma-Aldrich	C8487	WB
21	Anti-Integrin alphaV Antibody	Human Integrin αVβ3	LifeSpan BioSciences	LS-C15967	FACS
22	Bax Antibody (6A7)	N-terminal residues 12-24 to human Bax	Santa Cruz Biotechnology	sc-23959	WB
23	Bim Antibody (H-5)	Amino acids 4-195 of BimEL of human origin	Santa Cruz Biotechnology	sc-374358	WB
24	NOXA Antibody (114C307)	a fusion protein containing NOXA of human origin	Santa Cruz Biotechnology	sc-56169	WB
25	Smad2/3 Antibody	Endogenous levels of total Smad2/3 protein	Cell Signaling Technology	5678	WB
26	Phospho-Smad2 (Ser465/467)/Smad3 (Ser423/425) (D27F4) Rabbit mAb	Endogenous levels of Smad2 protein when phosphorylated at Ser465 and Ser467	Cell Signaling Technology	12005	WB
27	Monoclonal Anti-β-Actin antibody	Slightly modified β-cytoplasmic actin N-terminal peptide	Sigma-Aldrich	A5441	WB

Supplementary Table 3 Gene cloning and related primers

No.	primer	Sequence 5' to 3'	target	vector
1	IRES-GFP-f	cccccccccctaacgttact	IRES-GFP	pLVET-IRES-GFP
2	IRES-GFP-r	ttacttgtacagctcgtccatgc		
5	β-catenin^{4A}-f	tttgccaccatggctactcaagctgacctgatgg	β-catenin^{4A}	pLVET-β-catenin^{4A}-IRES-GFP
6	β-catenin^{4A}-r	gctaggacgcgcgcgtaagcaggtggtccagggatttccatttc tcgttcgat		
9	K-Ras^{V12}-f	agctttgtttaaacatgtacccatacgatgttcca	K-Ras^{V12}	pLVET-K-Ras^{V12}-IRES-GFP
10	K-Ras^{V12}-r	cgacgcgtaggtggtcccataattacacactttgtctttgac		
11	H-Ras^{V12}-f	agctttgtttaaacatgacggaatataagctggtggtg	H-Ras^{V12}	pLVET-H-Ras^{V12}-IRES-GFP
12	H-Ras^{V12}-r	cgacgcgtaggtggtccggagagcacacacttgcagct		
13	DogITGA6-PmoF	ccgctcgagaatttgtattgtagcagtatgtg	ITGA6-promoter	pGL3-ITGA6-promoter
14	DogITGA6-PmoR	cccaagcttcagttcaaaacttggtttctg		

Supplementary Table 4 Target sequences of CRISPR/Cas9

No.	name	gene	Type	target region	target sequence 5' to 3'
1	lentiCRISPR/*ITGA6*-KO1	*ITGA6*	gRNA	CDS exon 1	CGGATTGAATTTGACAACGA
2	lentiCRISPR/*ITGA6*-KO2	*ITGA6*	gRNA	CDS exon 2	GTACAAGCCTCCAGTTCTGT
3	lentiCRISPR/*FOSL1*-KO1	*FOSL1*	gRNA	CDS exon 1	CCGGCGCAGACCCCCACTTC
4	lentiCRISPR/*FOSL1*-KO2	*FOSL1*	gRNA	CDS exon 2	GGTCCTAGGACCACCTCCAG

Supplementary Table 5 Oligonucleotides used in this study

No.	Experiment	primer description	Sequence 5' to 3'
1	RT-qPCR	α2-integrin-f	CACATAGGCTCCCACACCTT
2	RT-qPCR	α2-integrin-r	CCACAATGTTGTCGTTCCAG
3	RT-qPCR	α3-integrin-f	CATCTACCACAGCAGCTCCA
4	RT-qPCR	α3-integrin-r	CTCCTCCCCATGGATTACCT
5	RT-qPCR	α5-integrin-f	GACGACACGGAGGACTTTGT
6	RT-qPCR	α5-integrin-r	TGTCTGAGCCATTGAGGATG
7	RT-qPCR	α6-integrin-f	AGTGGAGCTGTGGTTTTGCT
8	RT-qPCR	α6-integrin-r	AGACCTTCCCCGTCAAAAAT
9	RT-qPCR	αV-integrin-f	TCCAGGTGGAGCTTCTTTTG
10	RT-qPCR	αV-integrin-r	TTCTTAGAGTGACCTGGAGACC
11	RT-qPCR	β1-integrin-f	ATCCCAGAGGCTCCAAAGAT
12	RT-qPCR	β1-integrin-r	GCTGGAGCTTCTCTGCTGTT
13	RT-qPCR	β3-integrin-f	GACCTTTGAGTGTGGGGTGT
14	RT-qPCR	β3-integrin-r	TCTTCCGAGCATTCACACTG
15	RT-qPCR	β4-integrin-f	ACAGTCCCAAGAAACGGATG
16	RT-qPCR	β4-integrin-r	CCTTCACCGTGTAGCGGTAT
17	RT-qPCR	β5-integrin-f	AAGCCCATCTCCACACACTC
18	RT-qPCR	β5-integrin-r	AGGAGAAGGGGCTCTCAGTC
19	RT-qPCR	β6-integrin-f	TGAGACCAGGCAGTGAACAG
20	RT-qPCR	β6-integrin-r	CCGAGAGGTCCATGAGGTAA
21	RT-qPCR	β8-integrin-f	CGTGACTTCCGTCTTGGATT
22	RT-qPCR	β8-integrin-r	CCTTTCTGGGTGGATGCTAA
23	RT-qPCR	Snail1-f	CAAGATGCACATCCGAAGC
24	RT-qPCR	Snail1-r	GAGCAGGAAAACGGCTTCTC
25	RT-qPCR	Snail2-f	CGTTTTCCAGACCCTGGTTA
26	RT-qPCR	Snail2-r	TGACCTGTCTGCAAATGCTC
27	RT-qPCR	Zeb1-f	GAAGGTGATCCAGCAAATG
28	RT-qPCR	Zeb1-r	CTTCCGCATTTTCTTTTTGG
29	RT-qPCR	Zeb2-f	GCCATTATTTACCCCGAAGC
30	RT-qPCR	Zeb2-r	AGTAGGGGCAGGTCAGCAGT
31	RT-qPCR	Twist1-f	GCCGGAGACCTAGATGTCATT
32	RT-qPCR	Twist1-r	CACGCCCTGTTTCTTTGAAT
33	RT-qPCR	Twist2-f	CTCGGACAAGCTCAGCAAGA
34	RT-qPCR	Twist2-r	TGCAGGACCTGGTAGAGGAA
35	ChIP-qPCR	α6-integrin-promoter-f	GCCCAAGAACTCTTGCCAAT
36	ChIP-qPCR	α6-integrin-promoter-r	GGTCTAAAGCCTCCTGCCTC
